# Decoding Stability and Selectivity in Unnatural DNA Base Pairs with Spatially Separated Recognition Interfaces

**DOI:** 10.1021/acs.joc.6c01057

**Published:** 2026-09-10

**Authors:** Andreu Bermejo, Josep Maria Bofill, Jordi Poater

**Affiliations:** † Departament de Química Inorgànica i Orgànica & IQTCUB, 16724 Universitat de Barcelona, Martí i Franquès 1-11, 08028 Barcelona, Spain; ‡ ICREA, Passeig Lluís Companys 23, 08010 Barcelona, Spain

## Abstract

Unnatural base pairs (UBPs) with spatially separated recognition interfaces offer a promising route to expand the genetic alphabet, yet the origin of their enhanced stability remains unclear. Herein, we present a quantum chemical investigation of alkynylated purine–pyridazine UBPs, combining Kohn–Sham molecular orbital and energy decomposition analyses to characterize both base-pairing and π–π stacking interactions. The UBPs display slightly stronger base-pair interaction energies than the canonical G–C pair, arising from a favorable balance of electrostatic and dispersion interactions that compensate for the increased Pauli repulsion. Importantly, modeling of experimentally relevant DNA trimers reveals a quite good correlation between increasing UBP content, enhanced stacking interaction energies, and experimentally observed increases in melting temperature. This agreement highlights the central role of dispersion-driven stabilization and supports the reliability of the computational approach in reproducing the observed trends. These results provide key insights into the factors governing UBP stability and establish a foundation for the rational design of expanded genetic systems.

## Graphical abstract

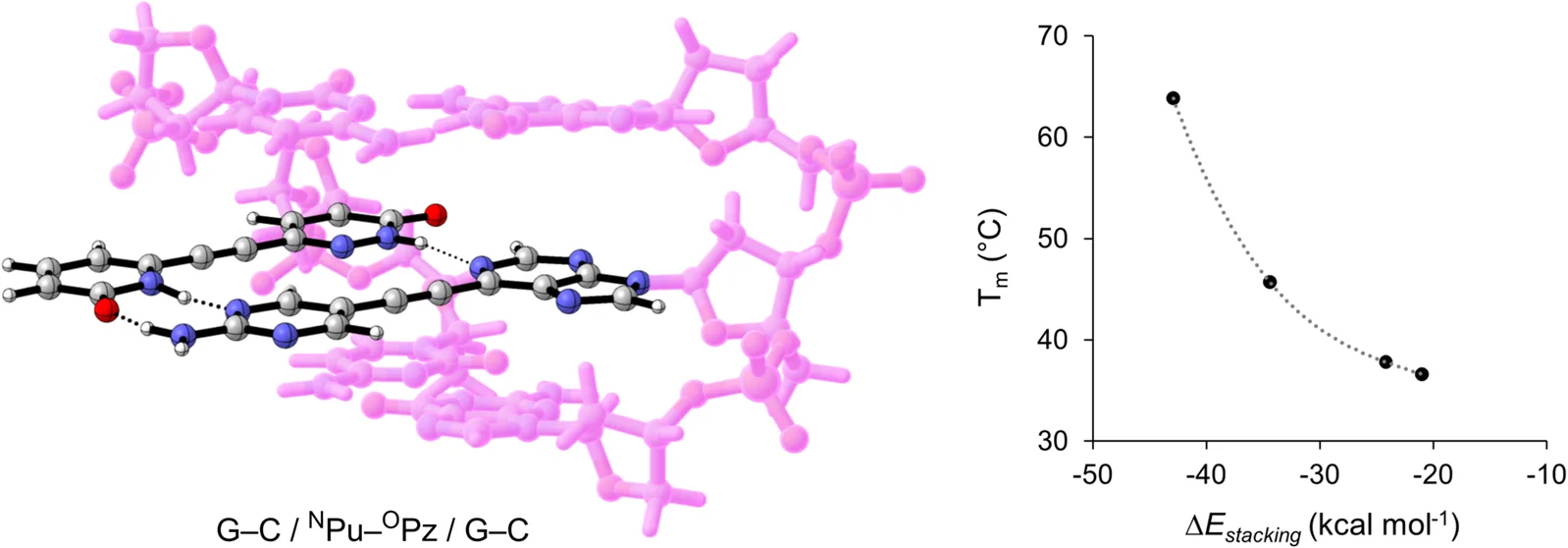

## Introduction

The ability of DNA to achieve highly specific molecular recognition and to encode information within its sequence has driven its study far beyond its canonical role in the *Central Dogma*, first articulated by Francis Crick in 1958.^1^ In recent decades, DNA has emerged as a versatile functional material with applications ranging from nanostructure-based diagnostics and drug delivery^2,3^ to its use as an alternative platform for data storage and molecular computing.^4,5^ These advances have positioned DNA as a central focus of both experimental and theoretical research.

In this context, the development of unnatural base pairs (UBPs) that can be incorporated into DNA without perturbing its structure—i.e., that are orthogonal to the natural A–T and G–C pairs (Figure 1A) and remain compatible with DNA polymerases—represents a powerful strategy to expand the functional scope of nucleic acids. Such systems not only enhance existing applications but also enable new ones, including the generation of XenoAptamers as alternatives to protein-based antibodies.^6–8^ Consequently, considerable effort has been devoted to expanding the genetic alphabet beyond its natural composition.^9^ A wide range of molecular recognition strategies has been explored to achieve this goal. These include modification^10^ or expansion^11,12^ of hydrogen-bond donor–acceptor patterns, replacement with metal-mediated coordination interactions,^13^ and even the complete elimination of direct interbase hydrogen bonding, in which π–π stacking becomes the dominant stabilizing force.^14–16^

**Figure 1:**
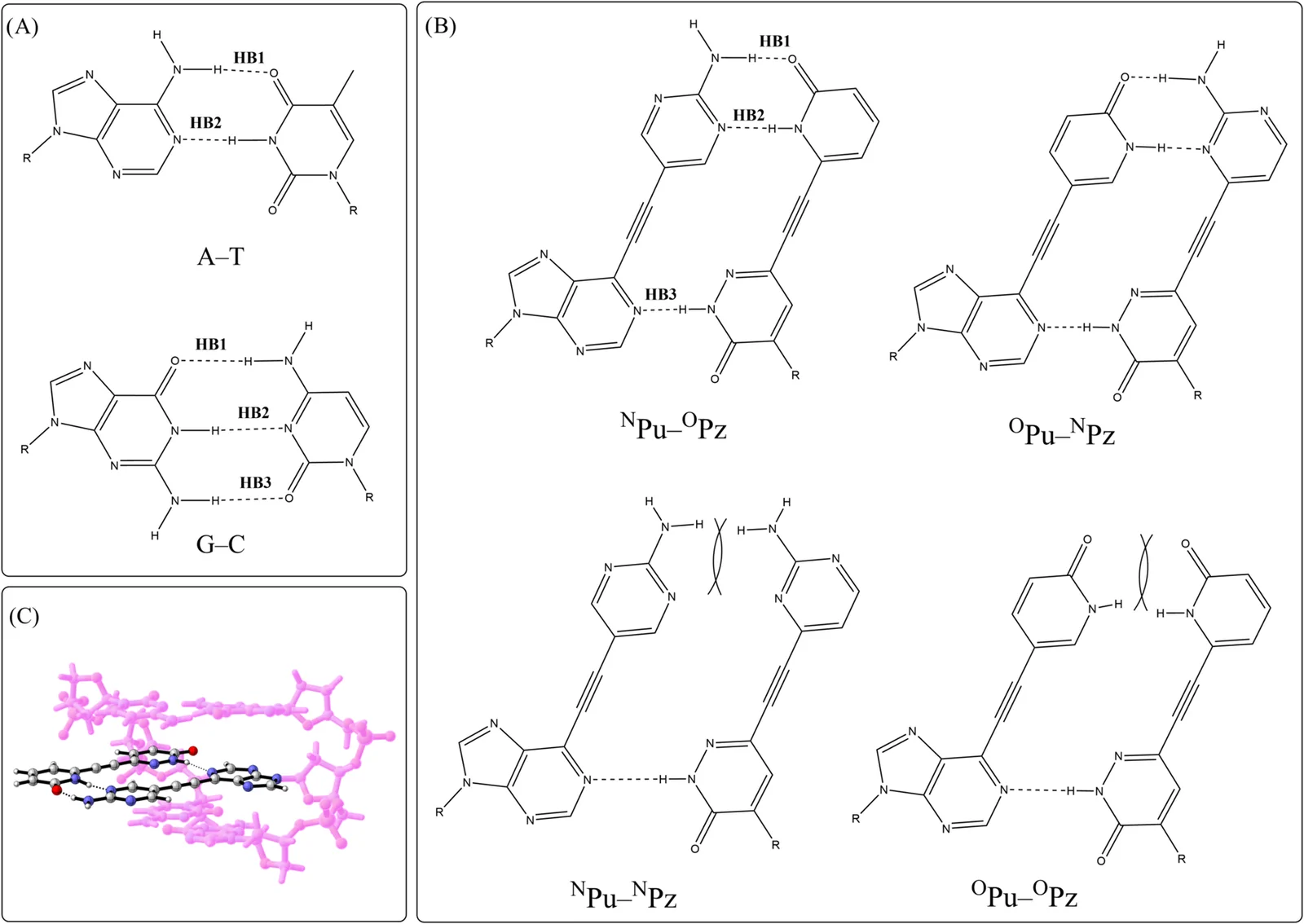
(A) Chemical structures of Watson-Crick base pairs (A–T and G–C). (B) Chemical structures of the UBPs featuring a spatially separated recognition interface: the complementary combinations ^N^Pu–^O^Pz and ^O^Pu–^N^Pz, and the non-complementary combinations ^N^Pu–^N^Pz and ^O^Pu–^O^Pz. (C) The unnatural DNA base pair ^N^Pu–^O^Pz within the B-DNA double helix showing major groove recognition driven by hydrogen bonding.

More recently, a novel design strategy based on the spatial separation of recognition elements via alkyne spacers has been introduced by Okamura and co-workers (Figure 1B).^17^ In this approach, alkynylated purines and pyridazines are functionalized with recognition units such as 2-pyridone and 2-aminopyrimidine, which exhibit low susceptibility to protonation or deprotonation under physiological conditions.^18,19^ This design enables reliable base recognition within the major groove of DNA (Figure 1C). The resulting base pairs (^N^Pu–^O^Pz and ^O^Pu–^N^Pz) form three hydrogen bonds, analogous to the natural G–C pair, but with distinct donor–acceptor patterns and spatial organization. Notably, two hydrogen bonds arise from the spatially separated recognition units, while the third is formed within the core scaffold and involves a nitrogen atom as the hydrogen-bond acceptor, in contrast to the oxygen-centered interaction in G–C. Experimental melting temperature measurements have shown that consecutive incorporation of these UBPs into DNA duplexes leads to significant thermal stabilization while preserving high sequence selectivity. This combination of stability and specificity highlights their potential for the development of functional oligonucleotides with enhanced performance.

Despite these promising results, a detailed theoretical understanding of the intermolecular forces governing these systems remains lacking. Elucidating the physical basis of their stability and selectivity is essential for guiding the rational design of next-generation UBPs. To address this gap, we constructed a simplified DNA model that excludes the polymerase active site and the sugar–phosphate backbone, thereby isolating the intrinsic interactions between nucleobases.^20–22^ Previous studies have shown that simplified B-DNA models lacking the backbone reproduce the essential trends governing hydrogen bonding, π–π stacking, and sequence selectivity, while allowing a physically transparent energy decomposition analysis.^20–23^ Herein, we present a comprehensive quantum chemical analysis of these systems, correlating theoretical insights with experimental observations to provide a detailed description of the interactions governing UBP formation. We anticipate that this work will contribute to the rational design and optimization of expanded genetic systems.

## Results and Discussion

To rationalize the origin of the selective and stable base pairing exhibited by these novel systems with spatially separated recognition interfaces, it is instructive to compare them with the canonical Watson–Crick G–C base pair. As noted above, these UBPs form three hydrogen bonds, analogous in number to G–C, but with two interactions located in the spatially separated recognition unit and one within the core scaffold. Despite this apparent similarity, the nature of the hydrogen bonds differs significantly. In the G–C pair, the three interactions correspond to O···H–N, N–H···N, and N–H···O (HB1, HB2, and HB3, respectively). In contrast, the ^N^Pu–^O^Pz pair exhibits N–H···O and double N···H–N interactions, whereas ^O^Pu–^N^Pz displays O···H–N, N–H···N, and N···H–N patterns for HB1, HB2, and HB3, respectively. Consequently, the hydrogen-bonding schemes are not equivalent: in terms of donor (D) and acceptor (A) character, G–C follows an ADD pattern, while ^N^Pu–^O^Pz and ^O^Pu–^N^Pz adopt DAA and ADA arrangements, respectively.

In the gas phase, both ^N^Pu–^O^Pz and ^O^Pu–^N^Pz exhibit base-pair interaction energies (−31.3 and −32.8 kcal mol^–1^, respectively) that are slightly stronger than that of the natural G–C pair (−30.2 kcal mol^–1^; Table 1). Analysis of the hydrogen-bond geometries shows that, relative to G–C, both UBPs present elongated HB1 distances and shortened HB3 distances. However, direct comparison of these distances must be made with caution, given the differing donor–acceptor roles involved in each interaction. Watson-Crick adenine-thymine (A–T) base pair has also been considered in all computations for comparison, although the discussion is based on G–C due to its closer resemblance to the UBPs under analysis in this work.

**Table 1:** Base-Pair Binding Energies (Δ*E*, in kcal mol^–1^) and H-Bond Distances (in Å) of Watson-Crick Base Pairs (A–T and G–C) and the UBPs with a Spatially Separated Recognition Interface (^N^Pu–^O^Pz and ^O^Pu–^N^Pz)^a^

				vacuo				water
System	Δ*E*	HB1	HB2	HB3	Δ*E*	HB1	HB2	HB3
A–T	–16.5	2.90	2.82		–8.7	2.92	2.87	
G–C	–30.2	2.76	2.91	2.90	–13.2	2.88	2.93	2.85
^N^Pu–^O^Pz	–31.3	2.80	2.93	2.85	–16.8	2.84	2.94	2.88
^O^Pu–^N^Pz	–32.8	2.83	2.91	2.84	–17.1	2.88	2.92	2.86

a Computed at the ZORA-BLYP-D3­(BJ)/TZ2P level of theory under C_s_ symmetry in the gas phase and with the aqueous environment modeled using the COSMO solvation model (ε = 78.4).

Upon inclusion of aqueous solvation, the interaction energies decrease and hydrogen-bond distances generally increase, consistent with stabilization of lone-pair electron density by the polar medium. Notably, under these conditions both UBPs retain significantly stronger interaction energies (−16.8 and −17.1 kcal mol^–1^ for ^N^Pu–^O^Pz and ^O^Pu–^N^Pz, respectively) than the G–C pair (−13.2 kcal mol^–1^, Table 1).^20,22–24^ In contrast to the gas-phase behavior, the trend in hydrogen-bond distances is reversed: HB1 becomes shorter, whereas HB3 becomes longer relative to G–C. A plausible explanation for this behavior is that solvation differentially stabilizes the donor and acceptor sites involved in each interaction. In particular, hydrogen bonds involving nitrogen atoms as acceptors (as in HB3 of the UBPs) are more strongly solvated than their oxygen-containing counterparts, leading to a reduction in their effective interaction strength and a concomitant elongation. Conversely, the hydrogen bonds located in the spatially separated recognition units (HB1), which involve less solvent-exposed or less polarizable environments, may experience a smaller degree of competitive solvation and thus become relatively strengthened, resulting in shorter distances. This differential solvation effect highlights the role of the local electronic environment and solvent accessibility in modulating hydrogen-bond geometry in these systems.

We have also analyzed potential mispairings between these novel bases, as previously considered by Okamura and co-workers (Figure 1B).^17^ Their molecular dynamics simulations revealed a broad conformational landscape accessible to these non-complementary combinations within a B-DNA environment. However, it is important to note that our results correspond to well-defined local minima on the potential energy surface, characterized in both the gas phase and aqueous solution under C_1_ symmetry. Both ^N^Pu–^N^Pz and ^O^Pu–^O^Pz systems exhibit a pronounced rotation of the recognition unit in the major groove of the alkynylated pyridazines relative to the core molecular plane (Figure 2). This distortion is quantified by the dihedral angle α (*X*_1_ *-X*_2_ *-X*_3_′*-X*_4_′), which adopts values of 50° and −30° in the gas phase for ^N^Pu–^N^Pz and ^O^Pu–^O^Pz, respectively, although the starting geometries were identical. In aqueous solution, only minor changes are observed (47° and −28°, respectively), indicating that this conformational feature is largely preserved upon solvation.

**Figure 2:**
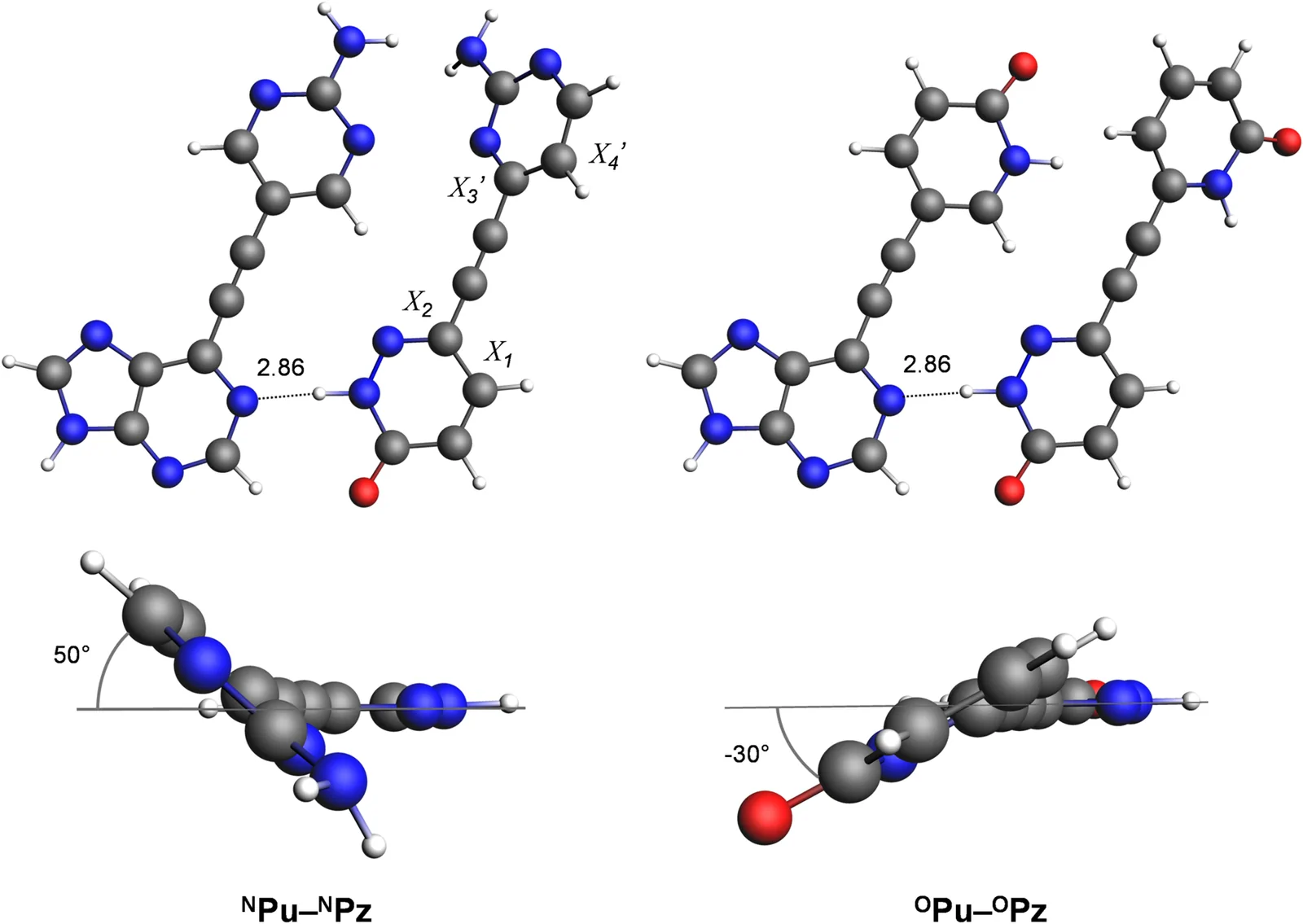
Optimized geometries of the non-complementary ^N^Pu–^N^Pz and ^O^Pu–^O^Pz pairs with the corresponding H-bond lengths (in Å) in the gas phase (top). Side views of the ^N^Pz and ^O^Pz are also shown with their corresponding dihedral angle α (*X*_1_-X_2_-X_3_′*-X*_4_′, bottom).

The computed base-pair interaction energies for these mismatched ^N^Pu–^N^Pz and ^O^Pu–^O^Pz pairs (−7.2 and −8.4 kcal mol^–1^, respectively; Table 2) are substantially weaker than those of the complementary ^N^Pu–^O^Pz and ^O^Pu–^N^Pz pairs. This reduction arises from the presence of a single hydrogen-bond interaction (HB3), in contrast to the three hydrogen bonds found in the complementary systems, leading to a marked decrease in overall stabilization. A similar energetic trend is observed in aqueous solution, where the interaction energies remain comparably weak. From a structural perspective, the remaining HB3 interaction exhibits a geometry comparable to that found in the complementary base pairs (Table 2), indicating that the reduced stability primarily reflects the loss of cooperative hydrogen-bonding interactions rather than a weakening of the individual bond itself.^25–28^ Overall, these results demonstrate that mismatched pairing between these bases is strongly disfavored, reinforcing the high selectivity of the complementary UBP systems, demonstrated at the base-pair level.

**Table 2:** Base-Pair Binding Energies (Δ*E*, kcal mol^–1^), H-Bond Distances (Å), and the Dihedral Angle α (°) of the Non-Complementary ^N^Pu–^N^Pz and ^O^Pu–^O^Pz Systems^a^

			vacuo			water
System	Δ*E*	HB3	α	Δ*E*	HB3	α
^N^Pu–^N^Pz	–16.0	2.86	50	–7.2	2.87	47
^O^Pu–^O^Pz	–17.5	2.86	–30	–8.4	2.88	–28

a Computed at the ZORA-BLYP-D3­(BJ)/TZ2P level of theory under C_1_ symmetry in the gas phase and with the aqueous environment modeled using the COSMO solvation model (ε = 78.4).

To gain deeper insight into the origin of the enhanced base-pair interaction energies of the UBPs relative to G–C, we have performed a quantitative Kohn–Sham molecular orbital analysis in combination with an energy decomposition analysis (EDA) (Table 3). The strain energies of the UBPs (2.9 kcal mol^–1^) are smaller than that of G–C (3.5 kcal mol^–1^), reflecting the increased structural rigidity imparted by the alkyne spacer. Consequently, the stronger base-pair binding in the UBPs arises from more favorable interaction energies (−34.2 and −35.7 kcal mol^–1^ for ^N^Pu–^O^Pz and ^O^Pu–^N^Pz, respectively, compared to −33.7 kcal mol^–1^ for G–C).

**Table 3:** Energy Decomposition Analysis (EDA) of the Base-Pair Binding Energies of Watson-Crick Base Pairs (A–T and G–C) and the UBPs with a Spatially Separated Recognition Interface (^N^Pu–^O^Pz, ^O^Pu–^N^Pz)^a^ ^,^ ^b^

System	Δ*E*	Δ*E*_strain_	Δ*E*_int_	Δ*E*_Pauli_	Δ*V*_elstat_	Δ*E*_oi_	Δ*E*_oi_^σ^	Δ*E*_oi_^π^	Δ*E*_disp_
A–T	–16.5	1.8	–18.3	38.0	–31.0	–20.1	–18.6	–1.5	–5.3
G–C	–30.2	3.5	–33.7	49.4	–46.6	–30.3	–26.0	–4.3	–6.2
^N^Pu–^O^Pz	–31.3	2.9	–34.2	60.3	–50.7	–32.3	–29.2	–3.1	–11.5
^O^Pu–^N^Pz	–32.8	2.9	–35.7	60.3	–51.2	–33.1	–29.6	–3.4	–11.8

a Computed at the ZORA-BLYP-D3­(BJ)/TZ2P level of theory under C_s_ symmetry in the gas phase.

b All values are given in kcal mol^–1^.

Decomposition of the interaction energy (Δ*E*_int_) reveals that the larger Pauli repulsion in the UBPs is effectively compensated by more attractive electrostatic and dispersion contributions, along with moderately enhanced orbital interactions (Table 3). The increased Pauli repulsion (+60.3 kcal mol^–1^ for the UBPs vs +49.4 kcal mol^–1^ for G–C) originates from the bulkier recognition units, which intensify closed-shell repulsion between the bases. However, this destabilizing term is outweighed by stronger attractive interactions.

In particular, the electrostatic contribution is more stabilizing by 4.1 and 4.6 kcal mol^–1^ for ^N^Pu–^O^Pz and ^O^Pu–^N^Pz, respectively (Table 3). This enhancement is closely related to the altered hydrogen-bond donor–acceptor patterns, which change from ADD in G–C to DAA and ADA in ^N^Pu–^O^Pz and ^O^Pu–^N^Pz, respectively. Consistently, Voronoi deformation density (VDD) charge analysis shows that in ^N^Pu–^O^Pz the charge distribution is effectively inverted relative to G–C, with ^N^Pu (+86 me) and ^O^Pz (−86 me) mirroring the magnitudes but reversing the signs of G (−86 me) and C (+86 me) (Figure 3). In ^O^Pu–^N^Pz, where only one hydrogen bond is inverted (ADA pattern), electron density is transferred from ^O^Pu (+68 me) to ^N^Pz (−68 me), again reflecting the modified donor–acceptor arrangement.

**Figure 3:**
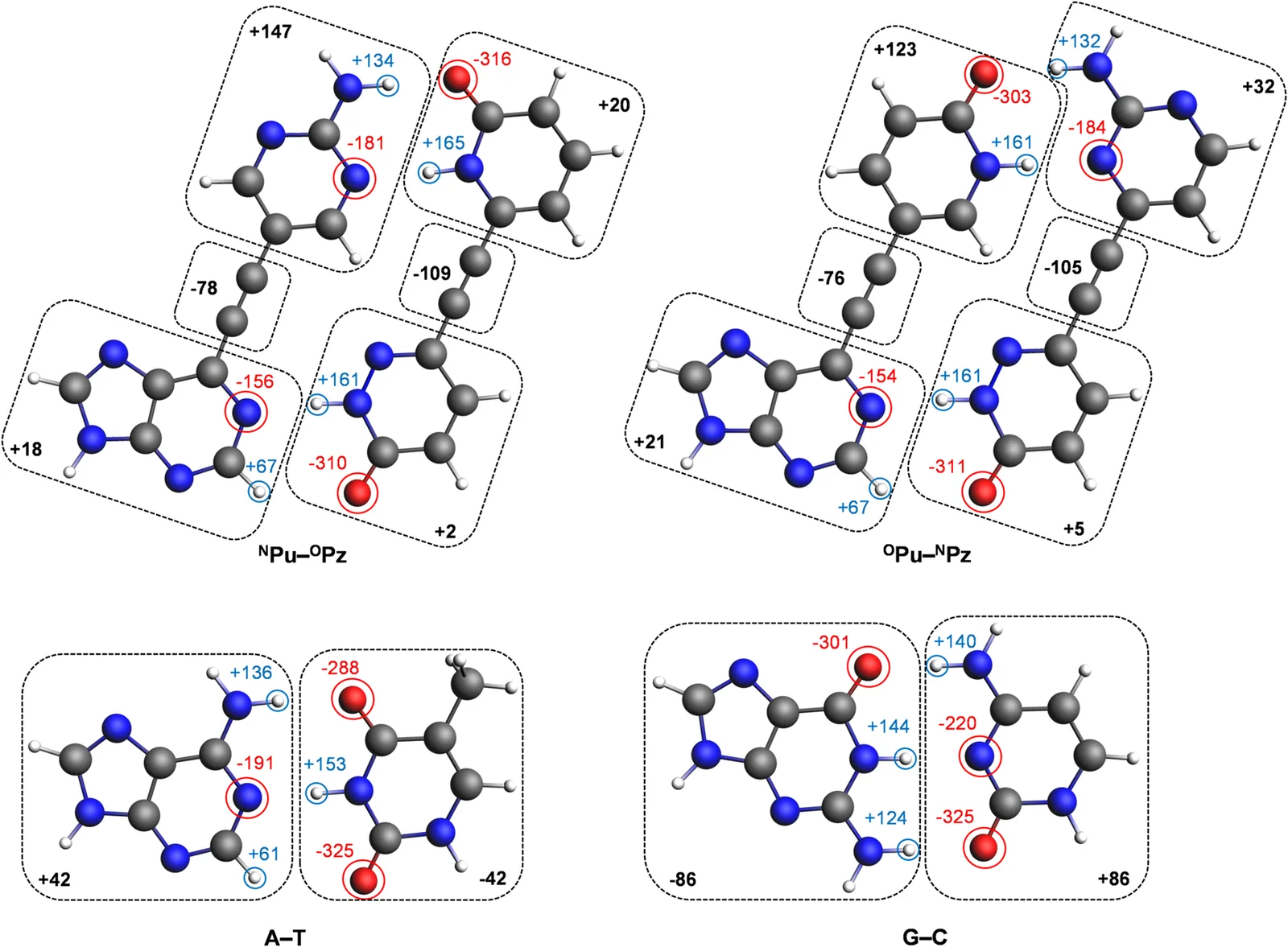
Optimized geometries of the complementary ^N^Pu–^O^Pz and ^O^Pu–^N^Pz pairs (top) and the natural A–T and G–C pairs (bottom). Selected Voronoi deformation density (VDD) charges are shown (in milli-e).

A key factor governing the electrostatic interactions in these UBPs is the presence of the acetylene linker, which withdraws electron density from both the core and recognition units. For example, the acetylene moieties in ^N^Pu and ^O^Pz carry charges of −78 and −109 me, respectively (Figure 3), with a more pronounced effect in the pyridazine fragment. This behavior is consistent with the low-lying π* orbitals of the alkyne, which facilitate electron density delocalization and reduce the electron density at both donor and acceptor sites.^26,27,29^

Among the attractive contributions, dispersion interactions play a particularly significant role in differentiating the UBPs from G–C. The dispersion term (Δ*E*_disp_) is substantially enhanced, being more stabilizing by 5.3 and 5.6 kcal mol^–1^ for ^N^Pu–^O^Pz and ^O^Pu–^N^Pz, respectively. This increase arises from the larger contact surface between the bases, which provides a greater number of atom–atom interactions while maintaining similar interfragment distances. Interaction region indicator (IRI) analysis further confirms the spatial localization of these stabilizing dispersion interactions (Section S2, Supporting Information).

Finally, orbital interactions (Δ*E*_oi_) provide a smaller but non-negligible contribution to the enhanced stability of the UBPs, being more stabilizing by 2.0 and 2.8 kcal mol^–1^ relative to G–C. Decomposition into σ and π components shows that the π contribution—primarily associated with intrafragment polarization—accounts for only 10–14% of Δ*E*_oi_ and is slightly less stabilizing in the UBPs (Table 3).^30–34^ In contrast, the σ component, dominated by interfragment charge transfer, is mainly responsible for the enhanced orbital interaction, contributing an additional stabilization of 3.2 and 3.6 kcal mol^–1^ for ^N^Pu–^O^Pz and ^O^Pu–^N^Pz, respectively.

As a reference system, in the G–C base pair, the σ interactions arise from one charge transfer from guanine to cytosine (HB1) and two in the opposite direction (HB2 and HB3). Specifically, HB1 involves donation from the guanine lone pair (HOMO) into the antibonding σ* N–H orbital of cytosine, whereas HB2 and HB3 correspond to donation from cytosine lone pairs into the σ* N–H orbitals of guanine (Figure S2).

A similar but reorganized pattern is observed in the UBPs. For ^N^Pu–^O^Pz, one charge transfer occurs from the pyridazine to the purine (HB1), while two occur in the reverse direction (HB2 and HB3). HB1 involves donation from the oxygen lone pair of ^O^Pz into the antibonding σ* N–H orbital of ^N^Pu, whereas HB2 and HB3 arise from nitrogen lone pairs of ^N^Pu donating into σ* N–H orbitals of ^O^Pz (Figure 4). Conversely, in ^O^Pu–^N^Pz, two charge transfers proceed from the purine to the pyridazine and one in the opposite direction, consistent with its ADA hydrogen-bonding pattern described above.

**Figure 4:**
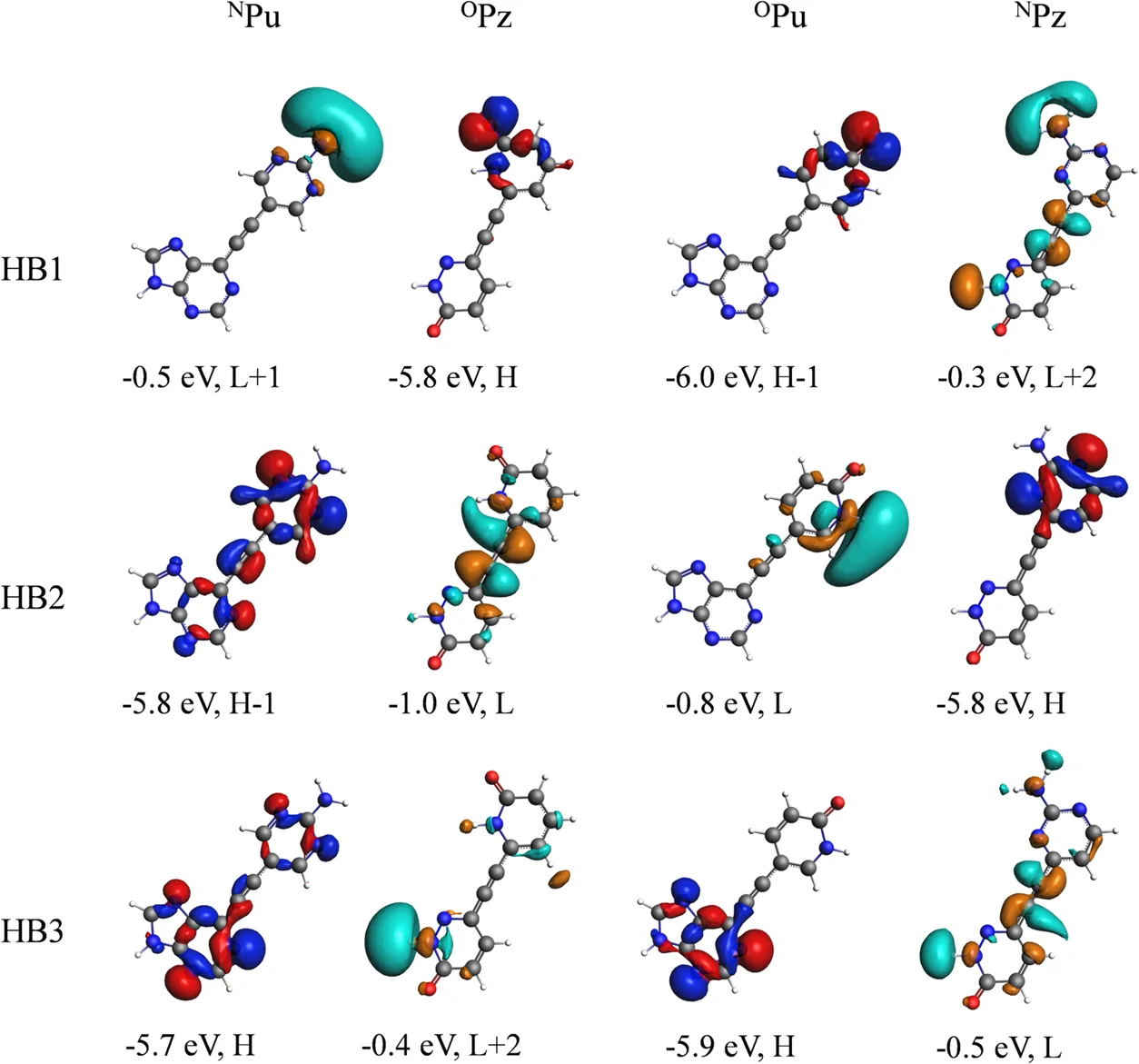
Main fragment molecular orbitals involved in the hydrogen bonding in ^N^Pu–^O^Pz (left) and ^O^Pu–^N^Pz (right). Energies in eV, H and L refer to HOMO and LUMO, respectively. Computed at ZORA-BLYP-D3­(BJ)/TZ2P level.

Overall, despite the differences in donor–acceptor arrangements relative to G–C, the enhanced orbital interactions in these UBPs originate from more effective overlap between fragment molecular orbitals involved in the hydrogen bonds, particularly in HB1 (Section S3, Supporting Information). Notably, the reduced nucleobase model employed and discussed here has been extensively validated in previous computational studies, which demonstrated that it reliably captures the intrinsic contributions of hydrogen bonding, π–π stacking, twist-angle preferences, and solvent effects while avoiding the additional complexity introduced by the sugar–phosphate backbone and explicit ions.^20–23^

The next step is to analyze π–π stacking interactions, which have been shown in previous studies to play a key role in the stability^35,36^ of base pairs within the DNA double helix. Although the average twist angle in natural B-DNA is well established to be approximately 36°, no theoretical studies to date have addressed the preferred twist angle for these UBPs. Thus, departing from the ^N^Pu–^O^Pz base pair as a reference, we examined the stacked systems ^N^Pu–^O^Pz/A–T, ^N^Pu–^O^Pz/G–C, and ^N^Pu–^O^Pz/^N^Pu–^O^Pz by varying the twist angle from 0° to 70° in 5° increments in the gas phase. The resulting interaction energy profiles (Figure 5) show that the minima for the mixed systems ^N^Pu–^O^Pz/A–T and ^N^Pu–^O^Pz/G–C occur at 45°, in close agreement with the ∼50° value reported for the G–C/G–C stack.^37^ As in that work, the energy difference between these minima and the canonical 36° twist angle of B-DNA is small (<1.0 kcal mol^–1^), indicating a relatively flat energy landscape and the absence of a strongly preferred orientation. Then, for the ^N^Pu–^O^Pz/^N^Pu–^O^Pz system, the minimum interaction energy is found at 25°. However, the energy difference relative to 36° remains similarly small (<1 kcal mol^–1^), again suggesting limited sensitivity of the stacking interaction to the precise twist angle. Overall, these results indicate that the canonical B-DNA twist angle is fully compatible with the stacking behavior of these UBPs, and this value was therefore adopted in subsequent analyses.

**Figure 5:**
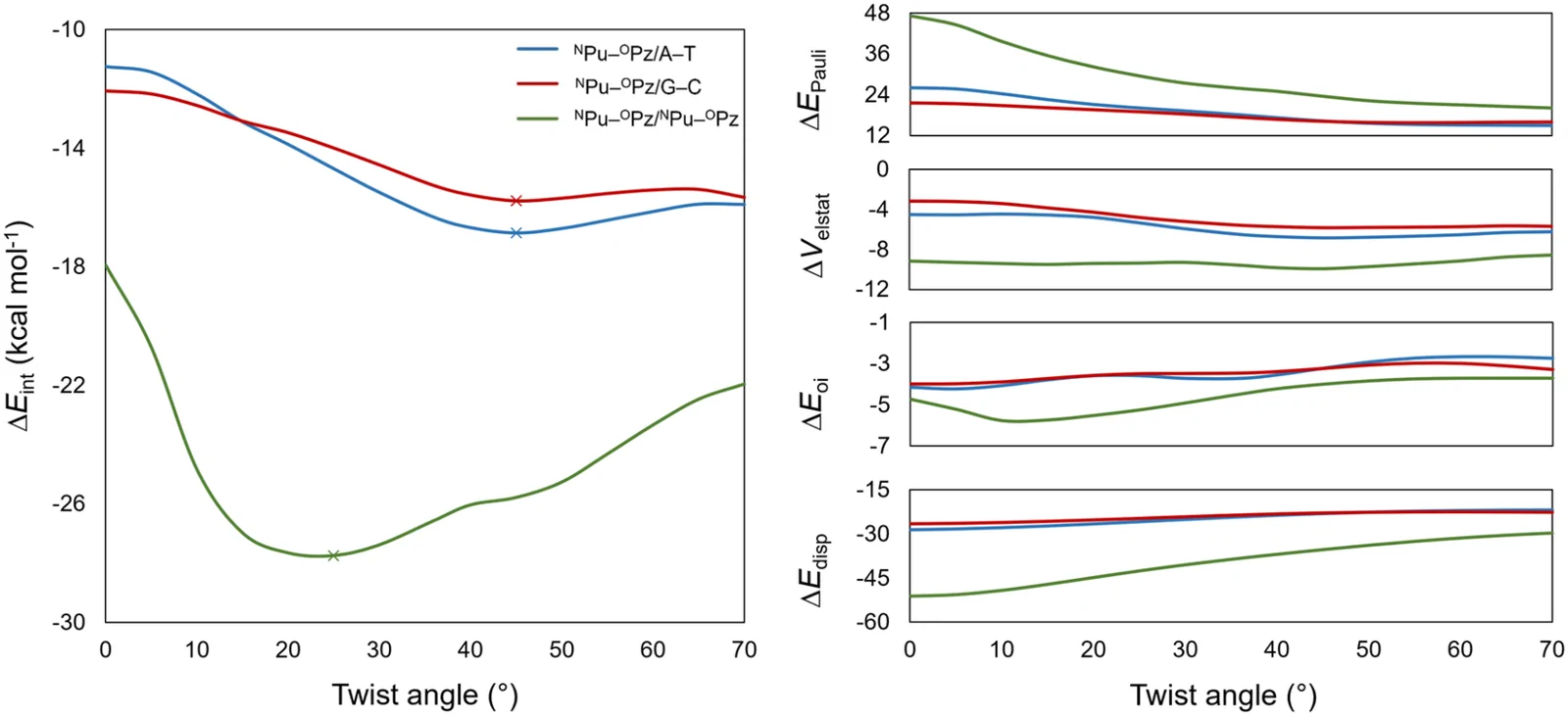
π–π stacking interaction energies (Δ*E*_int_) and the corresponding energy decomposition analysis (EDA) for the ^N^Pu–^O^Pz/A–T, ^N^Pu–^O^Pz/G–C, and ^N^Pu–^O^Pz/^N^Pu–^O^Pz systems, as a function of the twist angle (0-70°) in the gas phase. All values are given in kcal mol^–1^.

To rationalize the trends observed upon variation of the twist angle, an energy decomposition analysis was performed along the full rotational profile (numerical data are provided in Section S4 of the Supporting Information). Figure 5 illustrates the evolution of the different contributions to the π–π stacking interaction energy for the three systems studied. In all cases, dispersion constitutes the dominant stabilizing term. Its magnitude decreases progressively with increasing twist angle as the overlap between the aromatic surfaces diminishes, consistent with its R^–6^ dependence on intermolecular separation.^38^ Notably, in the ^N^Pu–^O^Pz/^N^Pu–^O^Pz system, the dispersion contribution at the equilibrium geometry is nearly twice as large (−42.6 kcal mol^–1^) as that in the mixed systems ^N^Pu–^O^Pz/A–T (−23.1 kcal mol^–1^) and ^N^Pu–^O^Pz/G–C (−22.9 kcal mol^–1^). This enhancement reflects the larger and more polarizable π-surface of the UBP, which increases the number and strength of interfragment dispersion interactions. This trend is further supported by reduced density gradient (RDG) analysis, which reveals more extensive regions of noncovalent interactions in the UBP/UBP stack (Section S5, Supporting Information). In contrast, the electrostatic and orbital contributions play a comparatively minor role and vary only modestly across the explored range of twist angles. These stabilizing terms collectively counterbalance the Pauli repulsion, which decreases with increasing twist angle as the aromatic rings move apart, thereby reducing unfavorable overlap between occupied orbitals. The interplay between the rapid decay of dispersion stabilization and the concurrent reduction of Pauli repulsion ultimately gives rise to the shallow energy profiles observed for all systems.

Finally, we turn to the experimentally studied 10-base-pair DNA duplexes reported by Okamura and co-workers, in which the central region is systematically modified by replacing one, two, or three consecutive Watson–Crick base pairs with natural or unnatural base pairs; accordingly, the identity of the base pairs flanking the central substitution(s) depends on the particular sequence considered.^17^ To rationalize the observed trends in thermal stability, a series of central trimers were modeled following the scheme shown in Figure 6. Computed π–π stacking interaction energies reveal that UBPs featuring spatially separated recognition interfaces exhibit stronger stacking interactions than their natural counterparts in the gas phase (Table 4). For example, substitution of the central G–C pair by ^N^Pu–^O^Pz in the G–C/G–C/G–C trimer increases the interaction energy from −25.4 to −33.5 kcal mol^–1^. Upon inclusion of solvation effects, a general attenuation of stacking interactions is observed, consistent with earlier trends in base-pair binding energies. Consequently, the difference between natural and unnatural systems becomes less pronounced (e.g., from −21.0 to −24.3 kcal mol^–1^ for the same trimers). For completeness, computed Gibbs energies follow the same trend (as well as the corresponding enthalpies and entropies), despite we must keep in mind that we are dealing with a model system involving frozen base pairs geometries, and thus these thermochemical corrections must be regarded as qualitative (Table S8).

**Figure 6:**
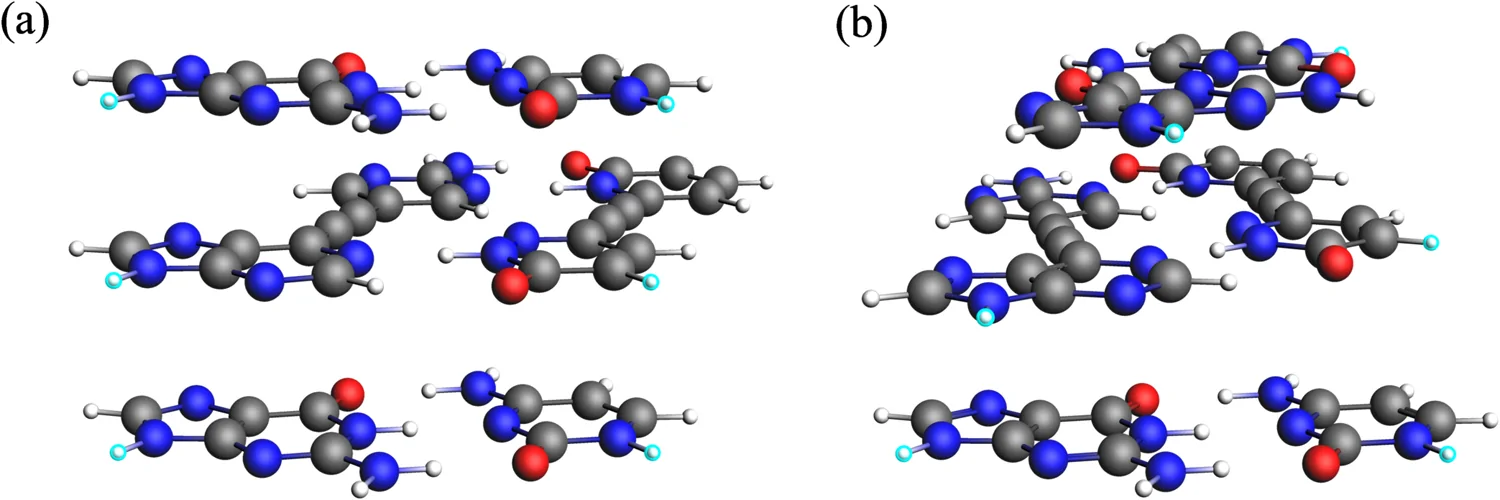
Preparation of the stacked G–C/^N^Pu–^O^Pz/G–C system. (a) Vertical separation of the base pairs by 3.4 Å. (b) Rotation of the middle base by 36° around the axis that runs through the center of mass of the upper and lower base, and subsequent rotation of the top base by 72° around the same axis, mimicking the effect of the backbone. The hydrogens highlighted in blue correspond to those introduced at the positions of the glycosidic bonds.

**Table 4:** π–π Stacking Interaction Energies Δ*E* (kcal mol^–1^) of Trimers, Including Watson-Crick and UBPs with a Spatially Separated Recognition Interface,^a^ and the Corresponding Experimental Melting Temperatures (*T*_m_, °C)^b^

System	Δ*E* (gas)	Δ*E* (water)	*T_m_*
G–C/A–T/G–C	–27.5	–21.2	31.8 ± 0.3
G–C/G–C/G–C	–25.4	–21.0	36.6 ± 0.3
G–C/^N^Pu–^O^Pz/G–C	–33.5	–24.2	37.8 ± 0.1
G–C/^O^Pu–^N^Pz/G–C	–33.9	–24.3	36.1 ± 0.6
G–C/^N^Pu–^O^Pz/^N^Pu–^O^Pz	–45.5	–34.4	45.7 ± 0.3
^N^Pu–^O^Pz/^N^Pu–^O^Pz/^N^Pu–^O^Pz	–54.4	–42.9	63.8 ± 0.4

a Computed at the ZORA-BLYP-D3­(BJ)/TZ2P level of theory in the gas phase, and with the aqueous environment modeled using the COSMO solvation model (ε = 78.4).

b Experimental *T*_m_ values obtained from UV melting measurements.^17^

More significantly, the stacking interaction energy increases markedly with the number of UBPs incorporated into the trimer. For instance, progressing from G–C/^N^Pu–^O^Pz/G–C to G–C/^N^Pu–^O^Pz/^N^Pu–^O^Pz and ultimately to ^N^Pu–^O^Pz/^N^Pu–^O^Pz/^N^Pu–^O^Pz results in stacking energies of −24.2, −34.4, and −42.9 kcal mol^–1^, respectively. This trend closely mirrors the experimentally observed increase in melting temperatures, which rise from 37.8 to 45.7 and to 63.8 °C across the same series (Section S6, Supporting Information). These results highlight the cumulative effect of enhanced π–π stacking interactions in UBP-rich sequences and provide a direct molecular-level explanation for their increased thermal stability. As pointed out above, the Watson–Crick adenine–thymine (A–T) base pair has been included in all calculations for completeness. Nevertheless, the discussion has been primarily centered on the G–C pair, given its closer structural and interactional similarity to the UBPs examined in this study. For these stacked systems, A–T has also been included (Table 4).

Finally, attempts to construct G–C/^N^Pu–^N^Pz/G–C and G–C/^O^Pu–^O^Pz/G–C trimers revealed pronounced nonplanarity of the mismatched pairs, which generates severe steric clashes with the neighboring Watson–Crick pairs and precludes a physically meaningful evaluation of their π–π stacking interactions within the constrained B-DNA model (Section S8 in the Supporting Information).

## Conclusions

In this work, we have provided a comprehensive theoretical analysis of unnatural base pairs (UBPs) featuring spatially separated recognition interfaces, with the aim of elucidating the origin of their enhanced stability and selectivity relative to canonical Watson–Crick base pairs. Through quantitative Kohn-Sham molecular orbital and energy decomposition analyses, we establish a detailed molecular-level understanding of both base-pairing and stacking interactions in these systems.

Noticeably, modeling of experimentally relevant DNA trimers reveals a quite good correlation between increased UBP content, enhanced stacking interaction energies, and experimentally observed increases in melting temperature. This finding underscores the cumulative nature of dispersion-driven stabilization in these systems and provides a direct theoretical explanation for their improved thermal stability.

Our results show that, despite differences in hydrogen-bond donor–acceptor patterns relative to G–C, the UBPs exhibit slightly stronger intrinsic base-pair interaction energies. This enhancement arises from a favorable balance of interaction components: although Pauli repulsion is increased due to the bulkier recognition units, it is more than compensated by stronger electrostatic and dispersion interactions, along with moderately enhanced orbital contributions. The presence of the acetylene linker plays a key role in modulating charge distribution and reinforcing electrostatic interactions, while also contributing to increased dispersion stabilization.

Investigation of π–π stacking interactions demonstrates that these UBPs are fully compatible with the canonical twist angle of B-DNA, with shallow energy profiles indicating minimal sensitivity to rotational variations. Notably, dispersion interactions dominate the stacking stabilization and are significantly enhanced in UBP-containing systems due to their larger and more polarizable π-surfaces.

Overall, this study highlights the critical role of dispersion and electrostatic interactions in governing both base pairing and stacking in UBPs with spatially separated recognition elements, previously synthesized by Okamura and co-workers.^17^ The insights obtained here provide a solid framework for the rational design of next-generation unnatural nucleobases with improved stability and functionality, thereby contributing to the expansion of the genetic alphabet and its applications in biotechnology and materials science.^39^

## Experimental Section

Calculations were performed using the Amsterdam Density Functional (ADF) program,^40^ using the dispersion-corrected density functional theory at the ZORA-BLYP-D3­(BJ)/TZ2P level of theory with the complete electron model for all atoms in both the gas phase and aqueous solution.^41–46^ Solvent effects were accounted for using the conductor-like screening model (COSMO), as implemented in the ADF, with a Delley-type cavity and the standard parameters for water (solvent radius 1.9 Å, dielectric constant 78.4).^47^ The chosen methodology has been extensively proven to be suitable for mechanistic analysis of DNA noncovalent interactions.^20,21,36,48,49^

The isolated nucleotide bases were fully optimized under C_s_ symmetry constraints, mimicking the nearly planar conformation observed in natural B-DNA. To evaluate the affinity between bases, the optimized structures were positioned to favor hydrogen-bond formation and then optimized under the same constraints. To support the validity of this model, additional calculations were performed without symmetry constraints, showing that C_s_-symmetric planar structures and C_1_-symmetric non-planar structures exhibit nearly identical stabilities (Section S1, Supporting Information). Base-pair binding energies were calculated as the energy difference between the optimized dimer and the optimized monomers

$$\Delta E=E_{\dim er\left[\mathrm{XY}\right]}-E_{monomer\left[X\right]}-E_{monomer\left[Y\right]}$$

The stacked trimers were constructed using the previously optimized base pairs following the scheme presented in Figure 6. The sugar-phosphate backbone was removed, and hydrogen atoms were inserted in its position, allowing a clear evaluation of the interactions between the nucleobases while maintaining a good balance between computational accuracy and efficiency.^22,48^ Consecutive base pairs were rotated 36°, which is the average rotation angle in B-DNA, with the aim of allowing a direct comparison to stacked Watson-Crick base pairs (Figure 6). π–π stacking interactions were calculated as the energy difference between the stacked system and the optimized dimers

$$\Delta E=E_{stacked\left[{X_{1}Y}_{1}/X_{2}Y_{2}/X_{3}Y_{3}\right]}-E_{\dim er\left[{X_{1}Y}_{1}\right]}-E_{\dim er\left[X_{2}Y_{2}\right]}-E_{\dim er\left[X_{3}Y_{3}\right]}$$

A Morokuma-Ziegler-type energy decomposition analysis (EDA) in the gas phase was carried out for both type of interactions, decomposing the interaction energy as Δ*E*_int_ = Δ*V*_elstat_ + Δ*E*_Pauli_ + Δ*E*_oi_ + Δ*E*_disp_, where Δ*V*_elstat_ corresponds to the classical electrostatic interaction, Δ*E*_Pauli_ to the repulsion between occupied orbitals, Δ*E*_oi_ to stabilizing orbital interactions (Δ*E*_*σ*_ + Δ*E*_π_), and Δ*E*_disp_ to the dispersion arising from electron correlation.^50,51^

The electron density distribution was analyzed using the Voronoi Deformation Density (VDD) method. This method assigns to each atom a VDD atomic charge defined as *Q*_*A*_^VDD^ = −∫_Voronoi_cell_of_*A*_ [ρ­(***r***) – ρ_promolecule_(***r***)]­d ***r***, where ρ­(***r***) is the electron density of the molecule and ρ_promolecule_(***r***) is the density of a fictitious promolecule, constructed as the superposition of the electron densities ρ_B_ of the non-interacting atoms: ρ_promolecule_(***r***) = Σ_B_ρ_B_ *(* **r** *)*. The Voronoi cell of the atom *A* is defined as the region of space bounded by the bond midplanes that are perpendicular to all bond axes connecting nucleus *A* and its neighboring nuclei.^52,53^ This parameter measures if charge flows away from (*Q*_*A*_^VDD^ > 0) or toward (*Q*_*A*_^VDD^ < 0) the Voronoi cell.

For completeness, to visualize regions where noncovalent interactions occur, the reduced density gradient (RDG) method was used as implemented in Multiwfn. The RDG function is defined as RDG­(***r***) = (1/(2­(3π^2^)^1/3^))·|∇ρ­(***r***)|/ρ­(***r***)^4/3^, where ρ is the electron density and ***r*** the position vector in three-dimensional real space. To distinguish between the different interactions, the function sign­(λ_2_)­ρ was used, where λ_2_ is the second largest eigenvalue of the electron density Hessian. Positive and negative values of sign­(λ_2_) indicates repulsive and attractive interactions, respectively, and interaction strength scales with ρ.^54,55^ And finally, to visualize both covalent and noncovalent interactions in selected systems, the interaction region indicator (IRI) method was used as implemented in Multiwfn. The IRI function is defined as IRI­(***r***) = |∇ρ­(***r***)|/(ρ­(***r***))^*a*^, where *a* is an adjustable parameter, typically set at 1.1. This method only differs from RDG by a constant prefactor.^56^

## Supplementary Material



## Data Availability

The data underlying this study are available in the published article and in its Supporting Information.

## Notes

Validation of the model used, interaction region indicator analysis, orbital interaction analysis, effect of the twist angle in π–π stacking interactions, reduced density gradient analysis, correlation between π–π stacking interactions and melting temperatures, and Cartesian coordinates of all systems under analysis together with their ADF electronic energies (PDF)
