# Supplementary material for: Decoding Stability and Selectivity in Unnatural DNA Base Pairs with Spatially Separated Recognition Interfaces
Source: J Org Chem. 2026 Sep 10;91(37):12512–21. doi: 10.1021/acs.joc.6c01057 (PMC13595904; doi:10.1021/acs.joc.6c01057)
Supplement: Supplementary file 1 [file jo6c01057_si_001.pdf]

SUPPORTING INFORMATION

Decoding Stability and Selectivity in Unnatural DNA Base Pairs with Spatially Separated Recognition Interfaces

Andreu Bermejo,[a] Josep Maria Bofill,[a,*] and Jordi Poater[a,b,*]

[a] Departament de Química Inorgànica i Orgànica & IQTCUB, Universitat de Barcelona, Martí i Franquès 1-11, 08028 Barcelona, Spain

[b] ICREA, Passeig Lluís Companys 23, 08010 Barcelona, Spain

CONTENTS

S1. Validation of the Model Used S3

S2. Interaction Region Indicator (IRI) Analysis S4

S3. Orbital Interaction Analysis S5

S4. Effect of the Twist Angle in π-π Stacking Interactions S10

S5. Reduced Density Gradient (RDG) Analysis S12

S6. Correlations between π-π stacking and melting temperatures S13

S7. Gibbs free energies of trimers S14

S8. Trimers of mismatched pairs S15

S9. Optimized Geometries in the Gas Phase S16

S10. Optimized Geometries in Water S30

S1. Validation of the Model Used

Base-pair bonding energies (ΔE) and H-bond distances (HB) for the natural and unnatural DNA base pairs were calculated and compared under C_s and C_1 symmetry constraints. The only notable difference is a 0.5 kcal mol^{-1} increase in the G–C base-pair bonding energy, attributed to the pyramidalization of the N2 amino group of guanine, as reported in previous studies.[1] H-bond distances show no significant differences.

System	ΔE (kcal mol^{-1})		HB_1 (Å)		HB_2 (Å)		HB_3 (Å)	
	C_s	C_1	C_s	C_1	C_s	C_1	C_s	C_1
A–T	-16.5	-16.5	2.90	2.90	2.82	2.83	-	-
G–C	-30.2	-29.7	2.76	2.76	2.91	2.90	2.90	2.90
^{N}Pu–^{O}Pz	-31.3	-31.3	2.80	2.80	2.93	2.93	2.85	2.85
^{O}Pu–^{N}Pz	-32.8	-32.8	2.83	2.83	2.91	2.91	2.84	2.84

Table S1: Base-pair bonding energies (ΔE, kcal mol^{-1}) and H-bond distances (Å) of Watson-Crick base pairs (A–T and G–C) and the complementary UBPs with a spatially-separated recognition interface (^{N}Pu–^{O}Pz, ^{O}Pu–^{N}Pz) computed at the BLYP-D3(BJ)/TZ2P level of theory under C_s and C_1 symmetry in the gas phase.

S2. Interaction Region Indicator (IRI) Analysis

The Interaction Region Indicator (IRI) provides a global view of the interactions (both covalent and noncovalent) in a system. Orange and green regions (IRI < 1.0) indicate the regions where chemical bond or weak interactions occur, while the blue regions (IRI > 1.0) correspond to large gradients of electron density or negligible electron density and are not chemically relevant. Red points where IRI = 0.0 correspond to critical points (CPs) in the Atoms in Molecule (AIM) theory[2], where the magnitude of the electron density gradient is zero. Accordingly, the (3, +1) critical points are observed at the center of the rings, while the (3, -1) critical points occur between the interacting atoms (the first value corresponds to the dimensionality of space, and the second to the sum of the signs of the eigenvalues of the Hessian of the electron density, evaluated at the critical point). Analysis of the results shows that the most notable difference between the WC and UBPs are the interactions of the alkyne spacers with the adjacent base.

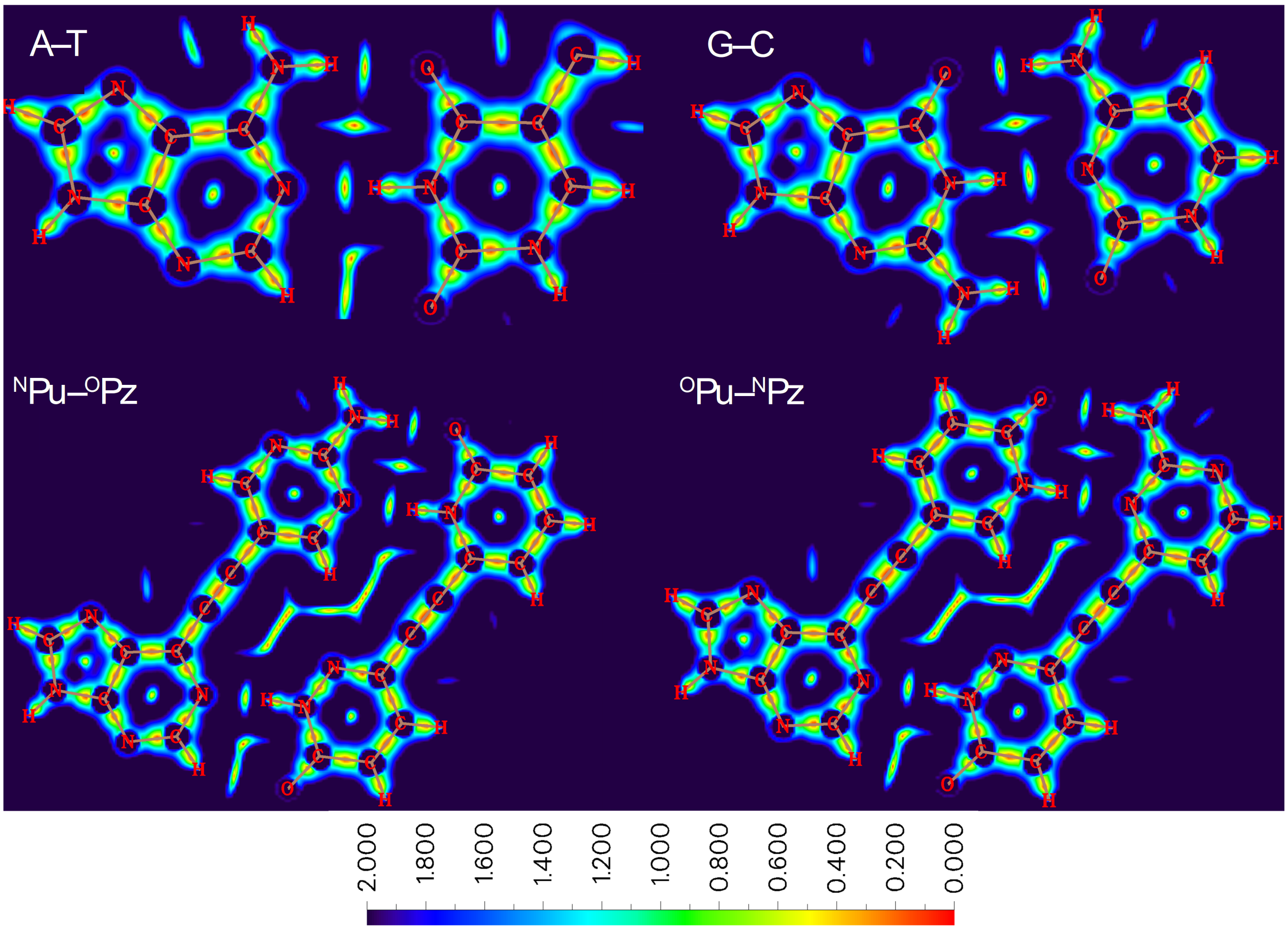

Figure S1. Interaction Region Indicator (IRI) for the Watson-Crick base-pairs (A–T and G–C, top) and for the UBPs with a spatially-separated recognition interface (^{N}Pu–^{O}Pz and ^{O}Pu–^{N}Pz, bottom).

S3. Orbital Interaction Analysis

For the natural G–C pair, the O···H–N hydrogen bond (HB_1) involves charge transfer from the σ_{HOMO} (0.06 electrons) of G to the σ_{LUMO} (0.01 electrons), σ_{LUMO+1} (0.01 electrons), σ_{LUMO+2} (0.01 electrons) and σ_{LUMO+3} (0.01 electrons) of C. The electron donation and acceptance are not identical in magnitude due to polarization of the σ-electron system.[3] The charge transfer in the N–H···N hydrogen bond (HB_2) goes from the σ_{HOMO-1} (0.05 electrons) of C to the σ_{LUMO} (0.04 electrons), σ_{LUMO+2} (0.02 electrons), and σ_{LUMO+3} (0.02 electrons) of G. Note that the gross populations of the acceptor orbitals exceeds the donated charge, as some of these orbitals are also involved in the N–H···O hydrogen bond (HB_3), where the charge transfer goes from the σ_{HOMO} (0.04 electrons) of C to the σ_{LUMO} (0.04 electrons), and σ_{LUMO+3} (0.02 electrons) of G. The accumulation of negative charge on the frontier atoms of C destabilizes the occupied orbitals, whereas the accumulation of positive charge in the frontier atoms of G stabilizes the unoccupied orbitals[4], in contrast to the UBPs, where no charge accumulation is observed.

G	Orbital energies (eV)	Gross populations (e^-)
σ_{LUMO+3}	0.82	0.02
σ_{LUMO+2}	-0.01	0.02
σ_{LUMO+1}	-0.37	0.00
σ_{LUMO}	-1.19	0.04
σ_{HOMO}	-5.64	1.94
σ_{HOMO-1}	-6.13	2.00
C	Orbital energies (eV)	Gross populations (e^-)
σ_{LUMO+3}	0.79	0.01
σ_{LUMO+2}	0.31	0.01
σ_{LUMO+1}	-0.46	0.01
σ_{LUMO}	-0.78	0.01
σ_{HOMO}	-5.45	1.96
σ_{HOMO-1}	-6.10	1.95
Significant Overlaps $\langle G \vert C \rangle$ for O···H–N (HB_1)		
$\langle \sigma_{HOMO} \vert \sigma_{LUMO+3} \rangle$	0.081	
$\langle \sigma_{HOMO} \vert \sigma_{LUMO+2} \rangle$	0.082	
$\langle \sigma_{HOMO} \vert \sigma_{LUMO+1} \rangle$	-0.052	
$\langle \sigma_{HOMO} \vert \sigma_{LUMO} \rangle$	-0.068	
Significant Overlaps $\langle G \vert C \rangle$ for N–H···N (HB_2) and N–H···O (HB_3)		
$\langle \sigma_{LUMO+3} \vert \sigma_{HOMO} \rangle$	-0.072	
$\langle \sigma_{LUMO+3} \vert \sigma_{HOMO-1} \rangle$	0.170	
$\langle \sigma_{LUMO+2} \vert \sigma_{HOMO-1} \rangle$	-0.235	
$\langle \sigma_{LUMO} \vert \sigma_{HOMO} \rangle$	-0.142	
$\langle \sigma_{LUMO} \vert \sigma_{HOMO-1} \rangle$	-0.218	

Table S2: Orbital energies (eV) and gross populations (e^-) of G and C, along with the significant orbital overlaps for the hydrogen-bonding interactions in the G–C pair.

For the ^{N}Pu–^{O}Pz base pair, the N–H···O hydrogen bond (HB_1) involves charge transfer from the σ_{HOMO} (0.05 electrons) of ^{O}Pz to the σ_{LUMO+1} (0.02 electrons) of ^{N}Pu. The charge transfer in the N···H–N hydrogen bond (HB_2) goes mainly from the σ_{HOMO} (0.04 electrons) of ^{N}Pu to the σ_{LUMO} (0.01 electrons) and σ_{LUMO+3} (0.01 electrons) of ^{O}Pz, and also from the σ_{HOMO-1} (0.03 electrons) of ^{N}Pu to the σ_{LUMO+4} (0.02 electrons) of ^{O}Pz. In the N···H–N hydrogen bond (HB_3) the charge transfer goes mainly from the σ_{HOMO} (0.04 electrons) of ^{N}Pu to the σ_{LUMO+1} (0.01 electrons) and σ_{LUMO+2} (0.04 electrons) of ^{O}Pz, from the σ_{HOMO-1} (0.03 electrons) of ^{N}Pu to the σ_{LUMO+2} (0.04 electrons) of ^{O}Pz, and also from the σ_{HOMO-2} (0.02 electrons) of ^{N}Pu to the σ_{LUMO+1} (0.01 electrons), σ_{LUMO+2} (0.04 electrons), and σ_{LUMO+3} (0.01 electrons) of ^{O}Pz.

^{N}Pu	Orbital energies (eV)	Gross populations (e^-)
σ_{LUMO+1}	-0.50	0.02
σ_{LUMO}	-0.55	0.00
σ_{HOMO}	-5.73	1.96
σ_{HOMO-1}	-5.78	1.97
σ_{HOMO-2}	-6.61	1.98
^{O}Pz	Orbital energies (eV)	Gross populations (e^-)
σ_{LUMO+4}	0.12	0.02
σ_{LUMO+3}	-0.07	0.01
σ_{LUMO+2}	-0.42	0.04
σ_{LUMO+1}	-0.70	0.01
σ_{LUMO}	-1.03	0.01
σ_{HOMO}	-5.75	1.95
σ_{HOMO-1}	-6.30	1.99
Significant Overlaps $\langle ^{N}Pu \mid ^{O}Pz \rangle$ for N–H···O (HB_1)		
$\langle \sigma_{LUMO+1} \mid \sigma_{HOMO} \rangle$	-0.113	
Significant Overlaps $\langle ^{N}Pu \mid ^{O}Pz \rangle$ for N···H–N (HB_2 and HB_3)		
$\langle \sigma_{HOMO} \mid \sigma_{LUMO+3} \rangle$	-0.119	
$\langle \sigma_{HOMO} \mid \sigma_{LUMO+2} \rangle$	-0.152	
$\langle \sigma_{HOMO} \mid \sigma_{LUMO+1} \rangle$	0.096	
$\langle \sigma_{HOMO} \mid \sigma_{LUMO} \rangle$	0.071	
$\langle \sigma_{HOMO-1} \mid \sigma_{LUMO+4} \rangle$	-0.153	
$\langle \sigma_{HOMO-1} \mid \sigma_{LUMO+2} \rangle$	-0.166	
$\langle \sigma_{HOMO-2} \mid \sigma_{LUMO+3} \rangle$	0.084	
$\langle \sigma_{HOMO-2} \mid \sigma_{LUMO+2} \rangle$	0.153	
$\langle \sigma_{HOMO-2} \mid \sigma_{LUMO+1} \rangle$	0.076	

Table S3: Orbital energies (eV) and gross populations (e^-) of ^{N}Pu and ^{O}Pz, along with the significant orbital overlaps for the hydrogen-bonding interactions in the ^{N}Pu–^{O}Pz pair.

For the ^{O}Pu–^{N}Pz base pair, the O···H–N hydrogen bond (HB_1) involves charge transfer mainly from the σ_{HOMO-1} (0.04 electrons) of ^{O}Pu to the σ_{LUMO} (0.02 electrons) and σ_{LUMO+2} (0.02 electrons) of ^{N}Pz. The charge transfer in the N–H···N hydrogen bond (HB_2) goes from the σ_{HOMO} (0.04 electrons) of ^{N}Pz to the σ_{LUMO} (0.05 electrons) of ^{O}Pu. In the N···H–N (HB_3) hydrogen bond the charge transfer goes mainly from the σ_{HOMO} (0.04 electrons) of ^{O}Pu to the σ_{LUMO} (0.02 electrons), σ_{LUMO+1} (0.01 electrons), σ_{LUMO+2} (0.02 electrons), and σ_{LUMO+3} (0.02 electrons) of ^{N}Pz, and also from the σ_{HOMO-2} (0.02 electrons) of ^{O}Pu to the σ_{LUMO} (0.02 electrons), σ_{LUMO+1} (0.01 electrons), σ_{LUMO+2} (0.02 electrons), and σ_{LUMO+3} (0.02 electrons) of ^{N}Pz.

^{O}Pu	Orbital energies (eV)	Gross populations (e^-)
σ_{LUMO+1}	-0.63	0.00
σ_{LUMO}	-0.77	0.05
σ_{HOMO}	-5.88	1.96
σ_{HOMO-1}	-5.97	1.96
σ_{HOMO-2}	-6.74	1.98
^{N}Pz	Orbital energies (eV)	Gross populations (e^-)
σ_{LUMO+3}	-0.11	0.02
σ_{LUMO+2}	-0.31	0.02
σ_{LUMO+1}	-0.39	0.01
σ_{LUMO}	-0.49	0.02
σ_{HOMO}	-5.80	1.96
σ_{HOMO-1}	-6.09	1.99
Significant Overlaps $\langle {}^{O}Pu \vert {}^{N}Pz \rangle$ for O···H–N (HB_1) and N···H–N (HB_3)		
$\langle \sigma_{HOMO} \vert \sigma_{LUMO+3} \rangle$	0.150	
$\langle \sigma_{HOMO} \vert \sigma_{LUMO+2} \rangle$	-0.122	
$\langle \sigma_{HOMO} \vert \sigma_{LUMO+1} \rangle$	0.072	
$\langle \sigma_{HOMO} \vert \sigma_{LUMO} \rangle$	0.126	
$\langle \sigma_{HOMO-1} \vert \sigma_{LUMO+2} \rangle$	-0.077	
$\langle \sigma_{HOMO-1} \vert \sigma_{LUMO} \rangle$	-0.054	
$\langle \sigma_{HOMO-2} \vert \sigma_{LUMO+3} \rangle$	0.122	
$\langle \sigma_{HOMO-2} \vert \sigma_{LUMO+2} \rangle$	0.100	
$\langle \sigma_{HOMO-2} \vert \sigma_{LUMO+1} \rangle$	0.062	
$\langle \sigma_{HOMO-2} \vert \sigma_{LUMO} \rangle$	0.101	
Significant Overlaps $\langle {}^{O}Pu \vert {}^{N}Pz \rangle$ for N–H···N (HB_2)		
$\langle \sigma_{LUMO} \vert \sigma_{HOMO} \rangle$	-0.212	

Table S4: Orbital energies (eV) and gross populations (e^-) of ^{O}Pu and ^{N}Pz, along with the significant orbital overlaps for the hydrogen-bonding interactions in the ^{O}Pu–^{N}Pz pair.

Visual inspection of the orbitals allows the assignment of frontier orbital interactions to specific hydrogen bonds.

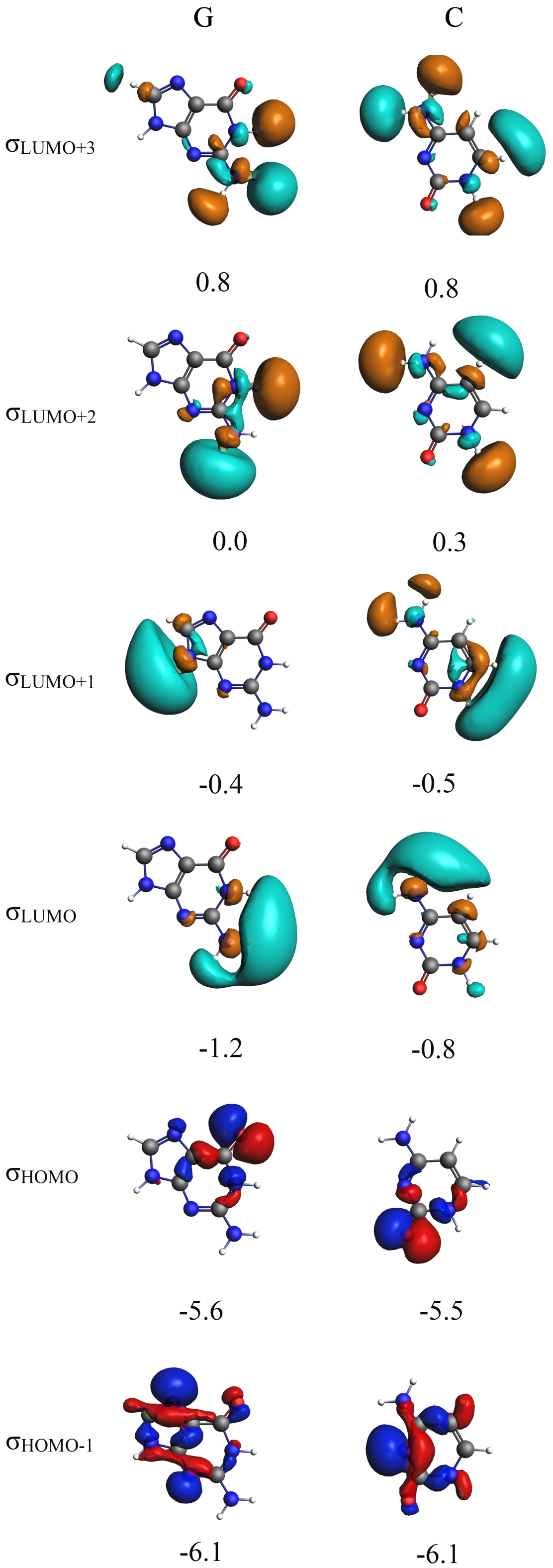

Figure S2. Isosurfaces (at 0.03 a.u.) of the unoccupied (σ_{LUMO+3}, σ_{LUMO+2}, σ_{LUMO+1}, and σ_{LUMO}) and occupied (σ_{HOMO} and σ_{HOMO-1}) orbitals of the G and C bases and the corresponding energies ε (in eV).

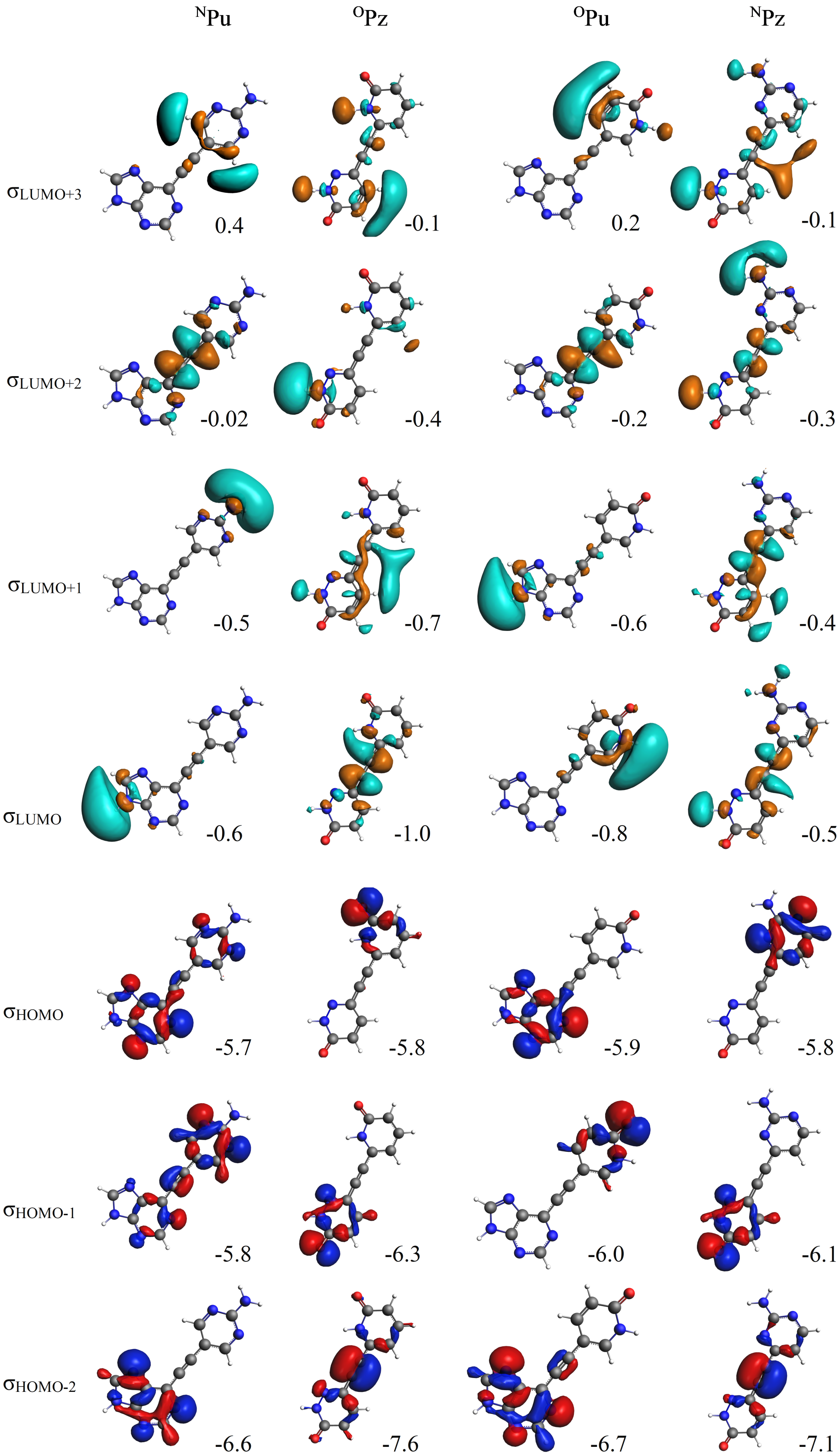

Figure S3. Isosurfaces (at 0.03 a.u.) of the unoccupied (σ_{LUMO+3}, σ_{LUMO+2}, σ_{LUMO+1} and σ_{LUMO}) and occupied (σ_{HOMO}, σ_{HOMO-1} and σ_{HOMO-2}) orbitals of the unnatural bases featuring a spatially-separated recognition interface (^{N}Pu, ^{O}Pz, ^{O}Pu, ^{N}Pz) and the corresponding energies ε (in eV).

S4. Effect of the Twist Angle in π-π Stacking Interactions

In the ^{N}Pu–^{O}Pz/A–T and ^{N}Pu–^{O}Pz/G–C systems, the minimum interaction energy is found at 45°, with a small difference relative to the experimentally observed 36° in natural B-DNA (<1 kcal mol^{-1}), suggesting that no preferred orientation is expected. In the case of the ^{N}Pu–^{O}Pz/^{N}Pu–^{O}Pz system, the minimum interaction energy occurs at 25°, but likewise, no significant difference is observed compared to 36° (<1.2 kcal mol^{-1}). Therefore, in all cases, the average twist angle observed in natural B-DNA is compatible with these interaction profiles. The final orientation is likely influenced by the sugar-phosphate backbone, as indicated in recent studies.[5]

^{N}Pu–^{O}Pz/A–T system					
Twist-angle (°)	ΔE_{int}	ΔV_{elstat}	ΔE_{Pauli}	ΔE_{oi}	ΔE_{disp}
0	-11.3	-4.5	26.0	-4.1	-28.6
5	-11.4	-4.6	25.6	-4.2	-28.3
10	-12.2	-4.5	24.2	-4.1	-27.8
15	-13.1	-4.6	22.5	-3.8	-27.3
20	-13.9	-4.8	21.1	-3.6	-26.6
25	-14.7	-5.4	20.0	-3.6	-25.8
30	-15.5	-5.9	19.2	-3.7	-25.0
36	-16.3	-6.5	18.0	-3.7	-24.2
40	-16.7	-6.7	17.2	-3.5	-23.6
45	-16.9	-6.8	16.2	-3.2	-23.1
50	-16.7	-6.8	15.6	-2.9	-22.6
55	-16.4	-6.7	15.2	-2.7	-22.3
60	-16.1	-6.5	15.1	-2.7	-22.0
65	-15.9	-6.3	15.0	-2.7	-21.9
70	-15.9	-6.2	14.9	-2.7	-21.8

Table S5: Energy decomposition analysis (EDA) of the π-π stacking interactions in the ^{N}Pu–^{O}Pz/A–T system as a function of the twist-angle (0-70°) in the gas phase. All values are given in kcal mol^{-1}.

^{N}Pu–^{O}Pz/G–C system					
Twist-angle (°)	ΔE_{int}	ΔV_{elstat}	ΔE_{Pauli}	ΔE_{oi}	ΔE_{disp}
0	-12.1	-3.2	21.6	-4.0	-26.6
5	-12.2	-3.2	21.4	-4.0	-26.4
10	-12.6	-3.4	20.8	-3.9	-26.1
15	-13.1	-3.8	20.2	-3.7	-25.7
20	-13.5	-4.3	19.6	-3.6	-25.3
25	-14.0	-4.8	19.0	-3.5	-24.7
30	-14.6	-5.2	18.3	-3.5	-24.2

36	-15.3	-5.6	17.4	-3.5	-23.6
40	-15.6	-5.7	16.8	-3.4	-23.3
45	-15.8	-5.8	16.2	-3.2	-22.9
50	-15.7	-5.8	15.9	-3.1	-22.7
55	-15.5	-5.8	15.8	-3.0	-22.6
60	-15.4	-5.7	15.9	-3.0	-22.5
65	-15.4	-5.7	16.0	-3.1	-22.6
70	-15.7	-5.7	16.0	-3.3	-22.7

Table S6: Energy decomposition analysis (EDA) of the π-π stacking interactions in the ^{N}Pu–^{O}Pz/G–C system as a function of the twist-angle (0-70°) in the gas phase. All values are given in kcal mol^{-1}.

^{N}Pu–^{O}Pz/^{N}Pu–^{O}Pz system					
Twist-angle (°)	ΔE_{int}	ΔV_{elstat}	ΔE_{Pauli}	ΔE_{oi}	ΔE_{disp}
0	-17.9	-9.2	47.1	-4.7	-51.2
5	-20.7	-9.3	44.5	-5.2	-50.7
10	-24.8	-9.4	39.6	-5.8	-49.2
15	-27.0	-9.5	35.4	-5.7	-47.2
20	-27.7	-9.4	32.1	-5.5	-44.9
25	-27.8	-9.4	29.5	-5.3	-42.6
30	-27.4	-9.3	27.3	-4.9	-40.5
36	-26.6	-9.6	25.8	-4.5	-38.3
40	-26.0	-9.8	24.9	-4.2	-36.9
45	-25.8	-9.9	23.5	-4.0	-35.4
50	-25.3	-9.7	22.2	-3.8	-33.9
55	-24.3	-9.4	21.4	-3.7	-32.5
60	-23.3	-9.2	20.9	-3.7	-31.4
65	-22.5	-8.8	20.5	-3.7	-30.5
70	-22.0	-8.6	20.0	-3.7	-29.7

Table S7: Energy decomposition analysis (EDA) of the π-π stacking interactions in the ^{N}Pu–^{O}Pz/^{N}Pu–^{O}Pz system as a function of the twist-angle (0-70°) in the gas phase. All values are given in kcal mol^{-1}.

S5. Reduced Density Gradient (RDG) Analysis

The reduced density gradient (RDG) is used to reveal the regions where noncovalent interactions occur. In these regions, the RDG exhibits relatively small values due to the low values of ρ and $\nabla\rho$. An RDG isovalue of 0.35 has been used, restricting the analysis to low-density regions (ρ < 0.05 a.u.). To distinguish between repulsive and attractive interactions, the sign of the second largest value of the electron density Hessian was used (λ_2), along with the value of ρ which is correlated with the strength of the weak interactions. The results clearly indicate a stronger dispersive interaction in the ^{N}Pu–^{O}Pz/^{N}Pu–^{O}Pz system compared to the ^{N}Pu–^{O}Pz/A–T and ^{N}Pu–^{O}Pz/G–C systems, as evidenced by a higher number of points around 0 in the RDG vs $sign(\lambda_2)\rho$ plot and a larger green isosurface in the 3D representation.

^{N}Pu–^{O}Pz/A–T (45°)

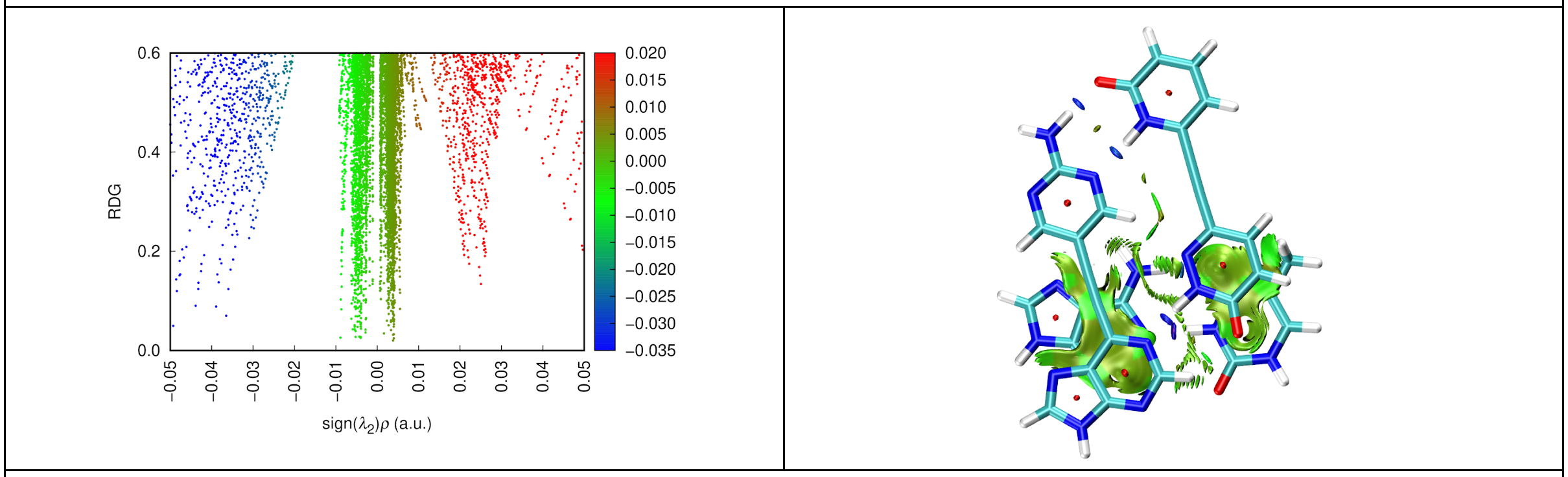

^{N}Pu–^{O}Pz/G–C (45°)

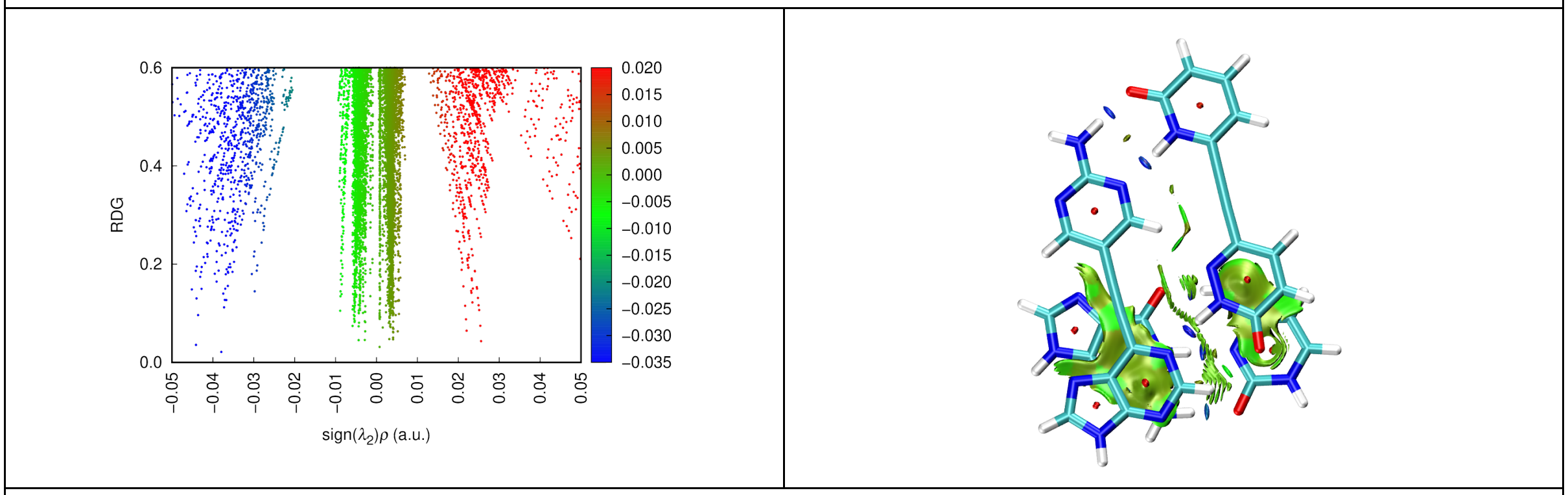

^{N}Pu–^{O}Pz/^{N}Pu–^{O}Pz (25°)

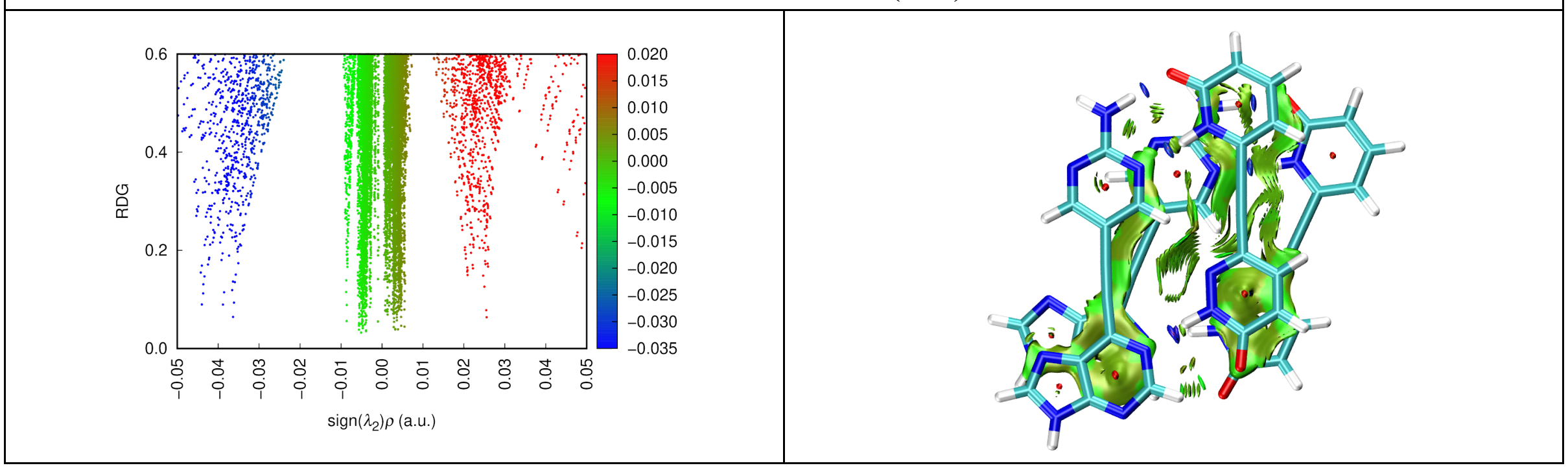

Figure S4. Reduced density gradient (RDG) analysis for the stacked ^{N}Pu–^{O}Pz/A–T (45°), ^{N}Pu–^{O}Pz/G–C (45°) and ^{N}Pu–^{O}Pz/^{N}Pu–^{O}Pz (25°) systems. For each system, both the RDG vs sign(λ_2)ρ plots and the 3D isosurfaces (isovalue = 0.35) are shown.

S6. Correlations between π-π stacking and melting temperatures

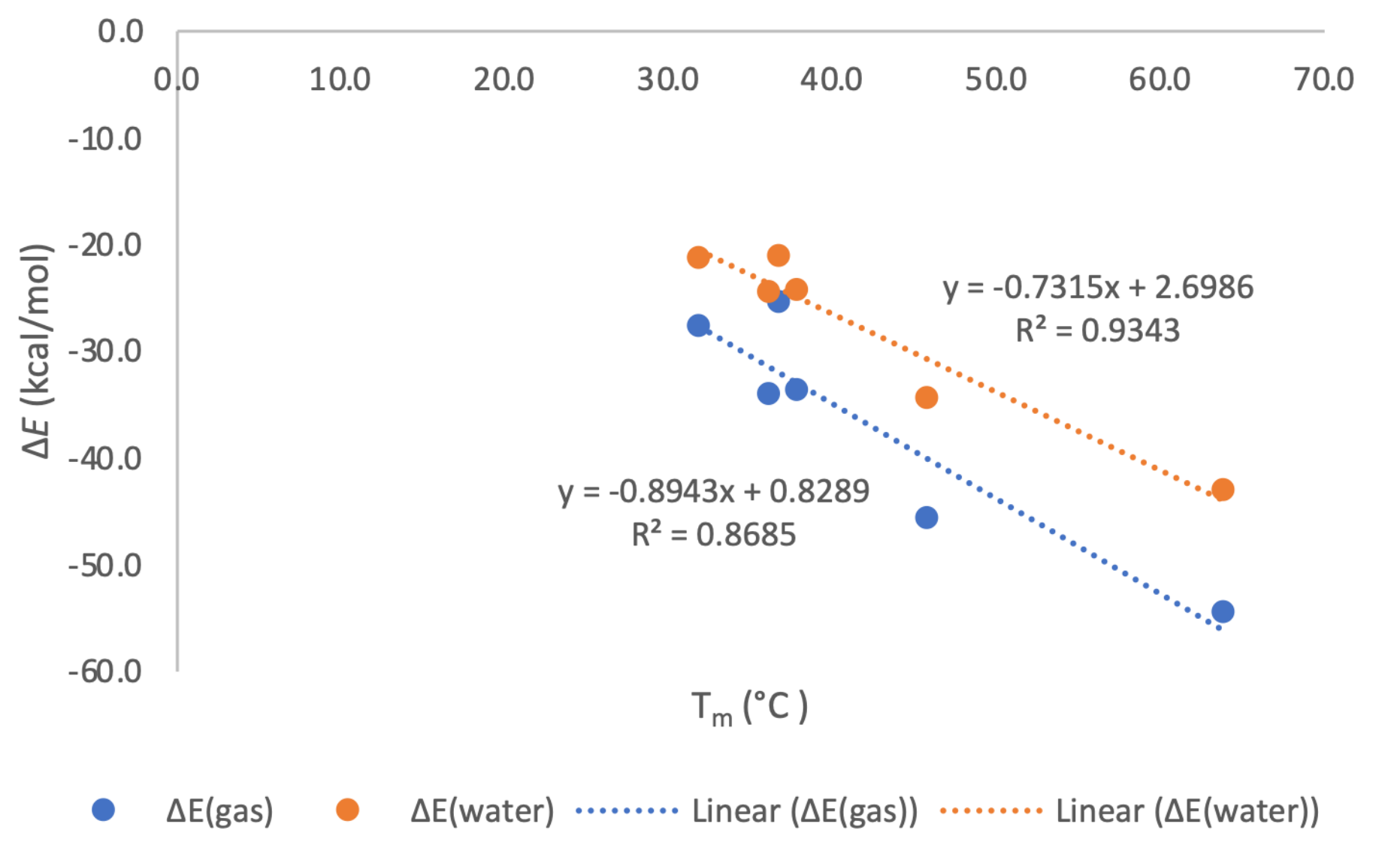

Figure S5. Correlation between the π-π stacking interaction energies Δ*E* (kcal mol^{-1}) of trimers, including Watson-Crick and UBPs with a spatially-separated recognition interface, both in vacuo and in water, and the experimental melting temperatures (T_m, °C). Stacking energies computed at the ZORA-BLYP-D3(BJ)/TZ2P level of theory in the gas phase, and with the aqueous environment modeled using the COSMO solvation model (ε = 78.4), and experimental T_m values obtained from UV melting measurements.

S7. Gibbs free energies of trimers

Table S8. π-π stacking interaction energies Δ*E* (kcal mol^{-1}) of trimers, together with thermal analysis, including Watson-Crick and UBPs with a spatially-separated recognition interface,[a] and the corresponding experimental melting temperatures (T_m, °C).[b]

**system**	**Δ*E* (gas)**	**Δ*H***	**-TΔ*S***	**Δ*G***	**T_m**
G–C/G–C/G–C	-25.4	-25.2	30.5	5.3	36.6±0.3
G–C/^{N}Pu–^{O}Pz/G–C	-33.5	-34.3	35.1	0.8	37.8±0.1
G–C/^{N}Pu–^{O}Pz/^{N}Pu–^{O}Pz	-45.5	-46.9	38.6	-8.3	45.7±0.3

[a] Computed at the ZORA-BLYP-D3(BJ)/TZ2P level of theory in the gas phase, and with the aqueous environment modeled using the COSMO solvation model (ε = 78.4). [b] Experimental T_m values obtained from UV melting measurements.[17]

S8. Trimers of mismatched pairs

We also attempted to construct and optimize the G–C/^{N}Pu–^{N}Pz/G–C and G–C/^{O}Pu–^{O}Pz/G–C trimers to evaluate the stacking interactions of the mismatched pairs within the same framework used for the complementary UBPs. However, unlike the complementary systems, both mispairs exhibit a pronounced departure from planarity, with dihedral angles of 49.6° and −30.0°, respectively. When these highly distorted mispairs are inserted between the planar Watson–Crick base pairs, severe steric clashes arise with the frozen neighboring G–C pairs, leading to unrealistic geometries (see image below) and stacking interaction energies that are dominated by artificial steric repulsion rather than intrinsic π–π interactions.

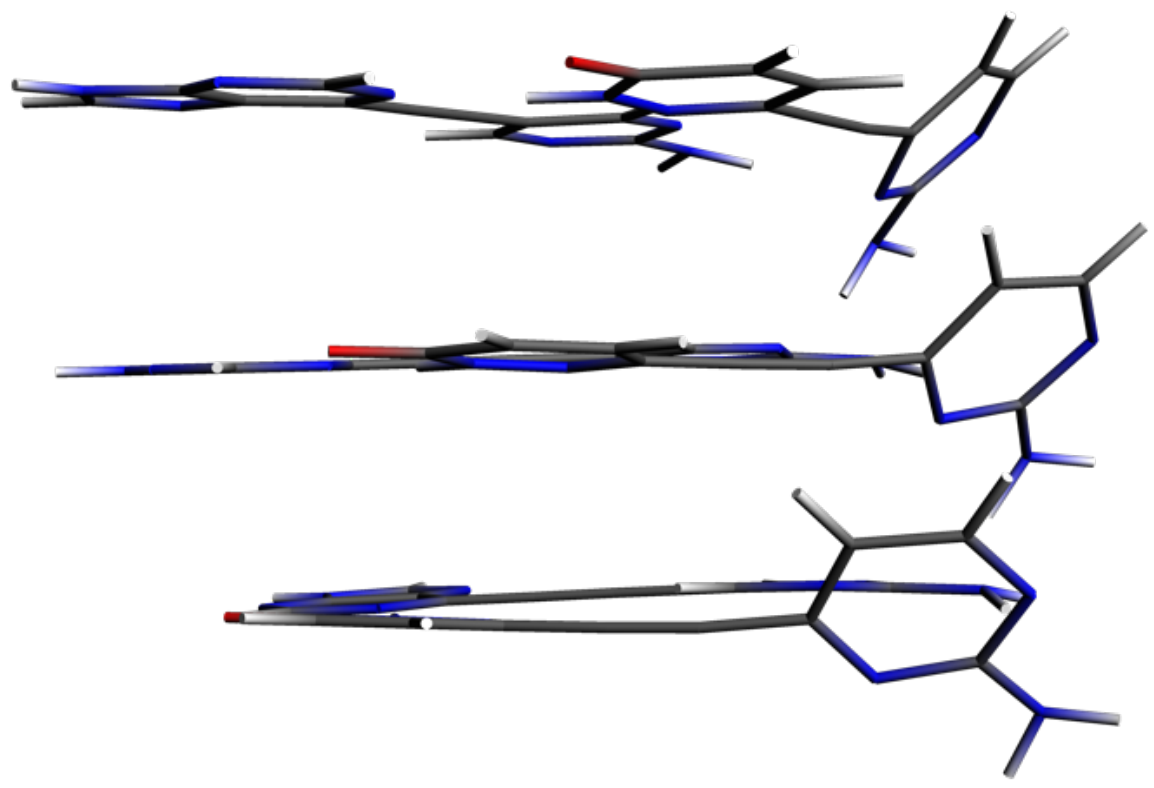

For this reason, we do not consider these trimer calculations to provide a physically meaningful description of stacking in the DNA duplex. Instead, we believe that the intrinsic selectivity is more appropriately assessed at the base-pair level, where the complementary UBPs are found to be substantially more stable than the corresponding mismatches. The distorted geometries adopted by the mismatches further support this conclusion, as they are inherently incompatible with efficient stacking within the B-DNA framework. Accordingly, selectivity is established primarily through complementary base pairing, while the trimer calculations are used to rationalize the enhanced thermal stability of the complementary UBP-containing duplexes.

In addition, already experimentally it is observed how the incorporation of just one mismatched pair does not affect the melting temperature. For instance, in case of ^{O}Pu–^{O}Pz, T_m is almost the same as for A-T (32º). What is needed to increase the melting temperature is the consecutive incorporation of complementary UBPs, whereas if they are mismatched pairs, T_m decreases, due to the steric repulsions referred above, giving rise to distortions in the helix.

S9. Optimized Geometries in the Gas Phase

Cartesian coordinates (Å) and ADF total bonding energies (in kcal mol^{-1}) of the WC base pairs optimized at BLYP-D3(BJ)/TZ2P level of theory in the gas phase.

A–T (-4490.7 kcal mol^{-1})

N	4.57183255	-1.43852731	0.00000000
C	4.09518869	-0.17316015	0.00000000
N	2.81375783	0.22237226	0.00000000
C	1.97355839	-0.82939634	0.00000000
C	2.32350107	-2.19056107	0.00000000
C	3.70811961	-2.49046906	0.00000000
N	0.58784311	-0.82575783	0.00000000
C	0.17956772	-2.15477095	0.00000000
N	1.19140388	-3.00209708	0.00000000
N	4.19663776	-3.74563787	0.00000000
H	4.85747099	0.60489205	0.00000000
H	0.00000000	0.00000000	0.00000000
H	-0.86902651	-2.42616576	0.00000000
H	3.54629097	-4.51971598	0.00000000
H	5.21191681	-3.91205125	0.00000000
N	7.36877342	-1.77356449	0.00000000
C	8.11440123	-0.60558684	0.00000000
N	9.49887252	-0.83431210	0.00000000
C	10.06585699	-2.09205480	0.00000000
C	9.31714560	-3.22488931	0.00000000
C	7.85771504	-3.08234688	0.00000000
O	7.07566923	-4.05000375	0.00000000
O	7.63580099	0.52452810	0.00000000
C	9.90464120	-4.60953621	0.00000000
H	6.31558431	-1.64584283	0.00000000
H	10.07500663	-0.00000000	0.00000000
H	11.15259232	-2.11265461	0.00000000
H	9.57063820	-5.17386146	0.87918469
H	10.99901520	-4.57177634	0.00000000
H	9.57063820	-5.17386146	-0.87918469

G–C (4396.4 kcal mol^{-1})

N	4.97119005	-1.05533672	0.00000000
O	4.34528524	3.53812458	0.00000000
N	4.55667268	1.23807420	0.00000000
C	4.06244004	-0.05043940	0.00000000
N	2.75810350	-0.33063542	0.00000000
C	1.98630422	0.77789430	0.00000000
C	2.37868353	2.13086291	0.00000000
C	3.77981851	2.42464605	0.00000000
N	0.60593209	0.81231267	0.00000000
C	0.23111076	2.15846856	0.00000000
N	1.26559360	2.96965052	0.00000000
H	5.98534901	-0.89040548	0.00000000
H	4.60944716	-1.99848779	0.00000000
H	5.58898402	1.37787815	0.00000000
H	0.00000000	0.00000000	0.00000000
H	-0.81025242	2.45627290	0.00000000
O	7.83318423	-0.57821431	0.00000000
N	7.07225634	3.96489646	0.00000000
N	7.42685680	1.69230122	0.00000000
C	9.36213639	3.17387907	0.00000000
C	8.23575027	0.59704963	0.00000000
N	9.63845619	0.82879603	0.00000000
C	10.17549177	2.08385156	0.00000000
C	7.93637707	2.93939270	0.00000000
H	9.77098431	4.17827316	0.00000000
H	10.22420135	-0.00000000	0.00000000
H	11.25945255	2.15100901	0.00000000
H	6.03848799	3.79721523	0.00000000
H	7.42143441	4.91346797	0.00000000

Cartesian coordinates (Å) and ADF total bonding energies (in kcal mol^{-1}) of the UBPs optimized at BLYP-D3(BJ)/TZ2P level of theory in the gas phase.

^{N}Pu–^{O}Pz (-7541.2 kcal mol^{-1})

N	1.52693277	-3.02113591	0.00000000
C	0.83309182	-4.17627349	0.00000000
N	-0.50701983	-4.32153312	0.00000000
C	-1.14136030	-3.14579832	0.00000000
C	-0.54476821	-1.85799606	0.00000000
C	0.86525107	-1.82407340	0.00000000
N	-2.50315578	-2.90281949	0.00000000
C	-2.66185395	-1.52113885	0.00000000
N	-1.51845350	-0.86452879	0.00000000
H	-3.64511408	-1.06589263	0.00000000
H	1.43864447	-5.08119693	0.00000000
H	-3.23053607	-3.60932364	0.00000000
C	1.63004120	-0.64269165	0.00000000
C	2.35176542	0.33894744	0.00000000
C	3.21564210	1.44769606	0.00000000
C	2.75137629	2.78805347	0.00000000
C	4.62154302	1.29181776	0.00000000
N	3.56186733	3.83635427	0.00000000
N	5.45976415	2.32544248	0.00000000
C	4.90340658	3.57999904	0.00000000
H	1.68018692	2.99091520	0.00000000
H	5.05343071	0.29304674	0.00000000
H	6.76696172	4.50584332	0.00000000
H	5.32737254	5.55488871	0.00000000
N	5.73780584	4.63035531	0.00000000
N	4.36096146	-3.35181614	0.00000000
C	4.75154713	-4.71487657	0.00000000
C	6.19656224	-4.89274998	0.00000000
C	7.02291313	-3.81184765	0.00000000
C	6.46652035	-2.48334694	0.00000000
N	5.14771814	-2.27536457	0.00000000
H	8.10477489	-3.92367376	0.00000000
O	3.89839385	-5.60833431	0.00000000
C	7.30487147	-1.33959922	0.00000000
H	3.32466945	-3.15717214	0.00000000
H	6.57087169	-5.91259688	0.00000000
C	8.05503015	-0.38106076	0.00000000
C	8.93042202	0.72923625	0.00000000
N	8.37174164	1.98996146	0.00000000
C	10.31148830	0.59379903	0.00000000
C	9.10776108	3.18618620	0.00000000
C	11.10954641	1.76213405	0.00000000
C	10.54276152	3.01444805	0.00000000
H	7.32988778	2.08076125	0.00000000
H	10.75008276	-0.39699443	0.00000000
H	12.19317796	1.66015817	0.00000000
H	11.14084771	3.92034329	0.00000000
O	8.51824334	4.28986767	0.00000000

^{O}Pu–^{N}Pz (-7541.2 kcal mol^{-1})

N	1.03131653	-2.44431050	0.00000000
C	2.01501057	-1.42235298	0.00000000
C	1.44454106	-0.08304515	0.00000000
C	0.09477428	0.08958302	0.00000000
C	-0.77517259	-1.05757011	0.00000000
N	-0.29372304	-2.30296252	0.00000000
H	-0.35112504	1.08149643	0.00000000
O	3.21536673	-1.71346537	0.00000000
C	-2.18611249	-0.91127938	0.00000000
H	1.38348796	-3.43942969	0.00000000
H	2.13939194	0.75198359	0.00000000
C	-3.39294980	-0.76054805	0.00000000
C	-4.80190986	-0.56545137	0.00000000
N	-5.58845495	-1.66983266	0.00000000
C	-5.34484968	0.73112335	0.00000000
C	-6.93697176	-1.46330616	0.00000000
C	-6.73977587	0.82083724	0.00000000

N	-7.54612053	-0.24097775	0.00000000
H	-8.73470695	-2.38832673	0.00000000
H	-4.70847205	1.60875701	0.00000000
H	-7.22937411	1.79576683	0.00000000
N	-7.73658014	-2.54750820	0.00000000
H	-7.35275751	-3.50430858	0.00000000
N	2.15706826	-5.05516913	0.00000000
C	3.50416754	-5.09386765	0.00000000
N	4.28716306	-6.19067952	0.00000000
C	3.57319780	-7.31993310	0.00000000
C	2.15833544	-7.43093866	0.00000000
C	1.43795514	-6.21846485	0.00000000
N	4.02870965	-8.62606041	0.00000000
C	2.90232337	-9.44153377	0.00000000
N	1.76951903	-8.76655470	0.00000000
H	2.98739315	-10.52169888	0.00000000
H	3.99561320	-4.12226114	0.00000000
H	5.00114583	-8.91360766	0.00000000
C	0.03329502	-6.13156876	0.00000000
C	-1.17579168	-5.98490576	0.00000000
C	-2.57361013	-5.81170705	0.00000000
C	-3.47968876	-6.92723843	0.00000000
C	-3.12804592	-4.53567540	0.00000000
C	-4.83303056	-6.73628854	0.00000000
N	-4.46735047	-4.35920093	0.00000000
C	-5.41180138	-5.41021621	0.00000000
H	-3.06222133	-7.93127883	0.00000000
H	-2.51074365	-3.64275904	0.00000000
O	-6.63308309	-5.15724122	0.00000000
H	-5.52994496	-7.56882254	0.00000000
H	-4.85619842	-3.38313515	0.00000000

^{N}Pu–^{N}Pz (-7540.0 kcal mol^{-1})

N	3.37037935	7.01858619	2.41648195
C	3.33642746	8.36374052	2.49017151
N	4.25375129	9.17275618	3.05672540
C	5.27521217	8.48771552	3.57826804
C	5.44210342	7.07849465	3.57748504
C	4.41963038	6.32854552	2.95867464
N	6.39135658	8.97444530	4.23517413
C	7.15918468	7.87005906	4.58858347
N	6.62675218	6.72329257	4.21470859
H	8.09707631	7.98120412	5.11969415
H	2.46218801	8.83067529	2.03877200
H	6.59265643	9.95178925	4.41529029
C	4.41751777	4.92384052	2.86929994
C	4.37677006	3.71030026	2.76410360
C	4.29918210	2.31516024	2.60693684
C	5.33657446	1.43874157	3.00841923
C	3.16429401	1.69139562	2.02609720
N	5.28977138	0.12421774	2.81311409
N	3.08474315	0.37943970	1.83691824
C	4.17001840	-0.35278809	2.20235393
H	6.22751157	1.83576062	3.49483135
H	2.31485610	2.29638947	1.71346443
H	3.43079556	-2.04516360	1.29771935
H	4.96943848	-2.22172981	2.11535274
N	4.12720783	-1.68708487	1.94760534
N	1.12174916	5.89396357	1.06279117
C	0.13184625	6.84700675	0.71858848
C	-1.03685830	6.24868173	0.09011563
C	-1.09828693	4.90334668	-0.11108912
C	0.00155052	4.06756595	0.29064187
N	1.09170942	4.57448547	0.86829131
H	-1.96512156	4.43812222	-0.57508269
O	0.30887539	8.04671894	0.95870256
C	-0.02755991	2.65740761	0.11342865
H	1.98333665	6.25338461	1.55008993
H	-1.84159151	6.91849167	-0.19966778
C	-0.03720813	1.44834320	-0.00804053
C	-0.02359510	0.02336321	-0.01802178
N	1.09299390	-0.58758126	-0.47257209
C	-1.11786279	-0.71393036	0.47771484
C	1.12460951	-1.92488971	-0.34671382

C	-0.97944991	-2.09998556	0.50398644
N	0.13848716	-2.72973371	0.11039374
H	2.25545589	-3.53166375	-0.87813279
H	-2.01537708	-0.21911396	0.83308888
H	-1.78924598	-2.73526124	0.86304524
N	2.32894587	-2.54231356	-0.66279084
H	2.93023960	-2.00112646	-1.27659568

^{O}Pu–^{O}Pz (-7511.7 kcal mol^{-1})

N	-3.71768878	6.01254704	0.05842256
C	-3.50768372	7.34416104	0.05006268
N	-4.42840167	8.31252929	-0.12532746
C	-5.65561067	7.81667869	-0.30422716
C	-6.02416249	6.44586101	-0.31885184
C	-4.98081752	5.51895178	-0.12276790
N	-6.83925357	8.50123125	-0.51248764
C	-7.83815760	7.54194965	-0.63863202
N	-7.39191008	6.30597973	-0.52899590
H	-8.87090766	7.82155437	-0.80977425
H	-2.47444488	7.65124386	0.20350376
H	-6.93984280	9.50922949	-0.55979595
C	-5.14859537	4.12132520	-0.09802906
C	-5.19520519	2.90523076	-0.05931803
C	-5.16492926	1.49779203	-0.00238838
C	-6.31760054	0.67187056	-0.25305333
C	-3.97022639	0.85942211	0.29977881
C	-6.23840907	-0.69163129	-0.21577510
N	-3.91141653	-0.49137493	0.35076443
C	-4.99445237	-1.38422278	0.07538472
H	-7.26096553	1.16174837	-0.48280213
H	-3.05513265	1.41443049	0.48083631
O	-4.79988006	-2.60178558	0.09785260
H	-7.10193932	-1.31915000	-0.41441171
H	-3.02472457	-0.95039842	0.55549798
N	-1.32953471	4.46250935	0.30266930
C	-0.18400807	5.27434237	0.50583587
C	1.04938510	4.50592612	0.60260498
C	1.02879890	3.15108328	0.48144118
C	-0.22097071	2.47140537	0.25816059
N	-1.37637264	3.13520843	0.17796146
H	1.94047432	2.56142815	0.54449563
O	-0.29509721	6.50125439	0.58337877
C	-0.27254832	1.05959786	0.11396163
H	-2.25559216	4.96277179	0.22521776
H	1.96779501	5.06216950	0.76754925
C	-0.28535360	-0.15157428	-0.01391151
C	-0.32206314	-1.56226157	-0.11708476
N	0.64161862	-2.28114683	0.56740229
C	-1.28140497	-2.25365798	-0.83544273
C	0.74841379	-3.70080302	0.60863531
C	-1.26269709	-3.67503458	-0.82235773
C	-0.30066755	-4.37408177	-0.14127899
H	1.34574637	-1.78222985	1.10585508
H	-2.02676125	-1.70864253	-1.40072020
H	-2.04154574	-4.20769758	-1.36289954
H	-0.28207479	-5.45941189	-0.12400161
O	1.66239262	-4.22696486	1.24706199

Cartesian coordinates (Å) and ADF total bonding energies (in kcal mol^{-1}) of the stacked systems optimized at BLYP-D3(BJ)/TZ2P level of theory in the gas phase.

G–C/A–T/G–C (-13311.0 kcal mol^{-1})

N	4.57183255	-1.43852731	0.00000000
C	4.09518869	-0.17316015	0.00000000
N	2.81375783	0.22237226	0.00000000
C	1.97355839	-0.82939634	0.00000000
C	2.32350107	-2.19056107	0.00000000
C	3.70811961	-2.49046906	0.00000000
N	0.58784311	-0.82575783	0.00000000
C	0.17956772	-2.15477095	0.00000000
N	1.19140388	-3.00209708	0.00000000

N	4.19663776	-3.74563787	0.00000000
H	4.85747099	0.60489205	0.00000000
H	0.00000000	0.00000000	0.00000000
H	-0.86902651	-2.42616576	0.00000000
H	3.54629097	-4.51971598	0.00000000
H	5.21191681	-3.91205125	0.00000000
N	7.36877342	-1.77356449	0.00000000
C	8.11440123	-0.60558684	0.00000000
N	9.49887252	-0.83431210	0.00000000
C	10.06585699	-2.09205480	0.00000000
C	9.31714560	-3.22488931	0.00000000
C	7.85771504	-3.08234688	0.00000000
O	7.07566923	-4.05000375	0.00000000
O	7.63580099	0.52452810	0.00000000
C	9.90464120	-4.60953621	0.00000000
H	6.31558431	-1.64584283	0.00000000
H	10.07500663	-0.00000000	0.00000000
H	11.15259232	-2.11265461	0.00000000
H	9.57063820	-5.17386146	0.87918469
H	10.99901520	-4.57177634	0.00000000
H	9.57063820	-5.17386146	-0.87918469
N	6.60546799	0.85026063	-3.40000000
O	3.39913155	-2.49803001	-3.40000000
N	4.92208327	-0.76150058	-3.40000000
C	5.27960996	0.57143148	-3.40000000
N	4.38907462	1.56478460	-3.40000000
C	3.11309847	1.12161745	-3.40000000
C	2.63528500	-0.20359192	-3.40000000
C	3.59614561	-1.26483395	-3.40000000
N	1.97612334	1.90513479	-3.40000000
C	0.88163594	1.03638624	-3.40000000
N	1.24174934	-0.22792750	-3.40000000
H	7.32899567	0.12072078	-3.40000000
H	6.86718213	1.82591301	-3.40000000
H	5.67506599	-1.48138173	-3.40000000
H	1.96337939	2.91846749	-3.40000000
H	-0.13588957	1.40755539	-3.40000000
O	8.64042440	-1.21797748	-3.40000000
N	5.35444730	-4.44616911	-3.40000000
N	6.97712306	-2.81602986	-3.40000000
C	7.67194753	-5.15218032	-3.40000000
C	8.27530436	-2.40540836	-3.40000000
N	9.27390017	-3.41738499	-3.40000000
C	8.97066795	-4.74840784	-3.40000000
C	6.65631164	-4.12443656	-3.40000000
H	7.41234441	-6.20506698	-3.40000000
H	10.23493204	-3.09116728	-3.40000000
H	9.80813648	-5.43987551	-3.40000000
H	4.61667169	-3.70287835	-3.40000000
H	5.07938194	-5.41882130	-3.40000000
N	3.52849073	0.26064229	3.40000000
O	5.72209191	-3.82344358	3.40000000
N	4.54117224	-1.83841331	3.40000000
C	3.38396035	-1.08648657	3.40000000
N	2.16403483	-1.62647301	3.40000000
C	2.19121352	-2.97694463	3.40000000
C	3.30391005	-3.84088446	3.40000000
C	4.61013345	-3.25499353	3.40000000
N	1.09469962	-3.81615206	3.40000000
C	1.58271337	-5.12552950	3.40000000
N	2.89642836	-5.17373573	3.40000000
H	4.44590671	0.72331780	3.40000000
H	2.68146429	0.81104040	3.40000000
H	5.45850436	-1.34473970	3.40000000
H	0.12702485	-3.51513525	3.40000000
H	0.91527786	-5.97855619	3.40000000
O	6.12433817	1.55688012	3.40000000
N	8.17910809	-2.56583589	3.40000000
N	7.13018792	-0.51883880	3.40000000
C	9.56671161	-0.57993165	3.40000000
C	7.14082375	0.84269401	3.40000000
N	8.41185379	1.47969708	3.40000000
C	9.58402784	0.77999742	3.40000000
C	8.27542046	-1.22826850	3.40000000
H	10.48784455	-1.15218876	3.40000000

H	8.39857750	2.49449952	3.40000000
H	10.50044469	1.36280207	3.40000000
H	7.24421137	-3.03781271	3.40000000
H	9.01915542	-3.12800464	3.40000000

G–C/G–C/G–C (-13214.6 kcal mol^{-1})

N	4.97119005	-1.05533672	0.00000000
O	4.34528524	3.53812458	0.00000000
N	4.55667268	1.23807420	0.00000000
C	4.06244004	-0.05043940	0.00000000
N	2.75810350	-0.33063542	0.00000000
C	1.98630422	0.77789430	0.00000000
C	2.37868353	2.13086291	0.00000000
C	3.77981851	2.42464605	0.00000000
N	0.60593209	0.81231267	0.00000000
C	0.23111076	2.15846856	0.00000000
N	1.26559360	2.96965052	0.00000000
H	5.98534901	-0.89040548	0.00000000
H	4.60944716	-1.99848779	0.00000000
H	5.58898402	1.37787815	0.00000000
H	0.00000000	0.00000000	0.00000000
H	-0.81025242	2.45627290	0.00000000
O	7.83318423	-0.57821431	0.00000000
N	7.07225634	3.96489646	0.00000000
N	7.42685680	1.69230122	0.00000000
C	9.36213639	3.17387907	0.00000000
C	8.23575027	0.59704963	0.00000000
N	9.63845619	0.82879603	0.00000000
C	10.17549177	2.08385156	0.00000000
C	7.93637707	2.93939270	0.00000000
H	9.77098431	4.17827316	0.00000000
H	10.22420135	-0.00000000	0.00000000
H	11.25945255	2.15100901	0.00000000
H	6.03848799	3.79721523	0.00000000
H	7.42143441	4.91346797	0.00000000
N	3.52849073	-0.26064229	-3.40000000
O	5.72209191	3.82344358	-3.40000000
N	4.54117224	1.83841331	-3.40000000
C	3.38396035	1.08648657	-3.40000000
N	2.16403483	1.62647301	-3.40000000
C	2.19121352	2.97694463	-3.40000000
C	3.30391005	3.84088446	-3.40000000
C	4.61013345	3.25499353	-3.40000000
N	1.09469962	3.81615206	-3.40000000
C	1.58271337	5.12552950	-3.40000000
N	2.89642836	5.17373573	-3.40000000
H	4.44590671	-0.72331780	-3.40000000
H	2.68146429	-0.81104040	-3.40000000
H	5.45850436	1.34473970	-3.40000000
H	0.12702485	3.51513525	-3.40000000
H	0.91527786	5.97855619	-3.40000000
O	6.12433817	-1.55688012	-3.40000000
N	8.17910809	2.56583589	-3.40000000
N	7.13018792	0.51883880	-3.40000000
C	9.56671161	0.57993165	-3.40000000
C	7.14082375	-0.84269401	-3.40000000
N	8.41185379	-1.47969708	-3.40000000
C	9.58402784	-0.77999742	-3.40000000
C	8.27542046	1.22826850	-3.40000000
H	10.48784455	1.15218876	-3.40000000
H	8.39857750	-2.49449952	-3.40000000
H	10.50044469	-1.36280207	-3.40000000
H	7.24421137	3.03781271	-3.40000000
H	9.01915542	3.12800464	-3.40000000
N	6.60546799	-0.85026063	3.40000000
O	3.39913155	2.49803001	3.40000000
N	4.92208327	0.76150058	3.40000000
C	5.27960996	-0.57143148	3.40000000
N	4.38907462	-1.56478460	3.40000000
C	3.11309847	-1.12161745	3.40000000
C	2.63528500	0.20359192	3.40000000
C	3.59614561	1.26483395	3.40000000
N	1.97612334	-1.90513479	3.40000000
C	0.88163594	-1.03638624	3.40000000

N	1.24174934	0.22792750	3.40000000
H	7.32899567	-0.12072078	3.40000000
H	6.86718213	-1.82591301	3.40000000
H	5.67506599	1.48138173	3.40000000
H	1.96337939	-2.91846749	3.40000000
H	-0.13588957	-1.40755539	3.40000000
O	8.64042440	1.21797748	3.40000000
N	5.35444730	4.44616911	3.40000000
N	6.97712306	2.81602986	3.40000000
C	7.67194753	5.15218032	3.40000000
C	8.27530436	2.40540836	3.40000000
N	9.27390017	3.41738499	3.40000000
C	8.97066795	4.74840784	3.40000000
C	6.65631164	4.12443656	3.40000000
H	7.41234441	6.20506698	3.40000000
H	10.23493204	3.09116728	3.40000000
H	9.80813648	5.43987551	3.40000000
H	4.61667169	3.70287835	3.40000000
H	5.07938194	5.41882130	3.40000000

G–C/^{N}Pu–^{O}Pz/G–C (-16367.5 kcal mol^{-1})

N	4.49675606	1.66092128	0.00000000
C	4.08556650	0.37769050	0.00000000
N	2.81422158	-0.07028460	0.00000000
C	1.92773825	0.92915896	0.00000000
C	2.21390905	2.31928955	0.00000000
C	3.57877754	2.67487234	0.00000000
N	0.54647006	0.85416665	0.00000000
C	0.07590335	2.16290401	0.00000000
N	1.03877555	3.06366951	0.00000000
H	-0.98542624	2.38114497	0.00000000
H	4.88207390	-0.36470859	0.00000000
H	0.00000000	0.00000000	0.00000000
C	4.05303118	3.99988188	0.00000000
C	4.53105457	5.12059361	0.00000000
C	5.11838295	6.39756338	0.00000000
C	4.35980477	7.59617086	0.00000000
C	6.52266165	6.56743619	0.00000000
N	4.90899138	8.80208311	0.00000000
N	7.10220035	7.76540514	0.00000000
C	6.27360050	8.85941986	0.00000000
H	3.27061377	7.54860508	0.00000000
H	7.17157811	5.69395009	0.00000000
H	7.87593978	10.18702456	0.00000000
H	6.23454293	10.87892744	0.00000000
N	6.84559113	10.07280239	0.00000000
N	7.33127993	1.98732974	0.00000000
C	8.02332540	0.74976602	0.00000000
C	9.47071273	0.90717484	0.00000000
C	10.02788061	2.14845224	0.00000000
C	9.18233083	3.31444223	0.00000000
N	7.85092171	3.21521674	0.00000000
H	11.10663545	2.28708103	0.00000000
O	7.39718555	-0.31516879	0.00000000
C	9.73680405	4.61964370	0.00000000
H	6.27794079	1.93974736	0.00000000
H	10.06839916	0.00000000	0.00000000
C	10.24779244	5.72437213	0.00000000
C	10.84597644	7.00548347	0.00000000
N	10.01370423	8.10497189	0.00000000
C	12.22140291	7.18957407	0.00000000
C	10.45655459	9.43784916	0.00000000
C	12.73102694	8.50949331	0.00000000
C	11.89278930	9.59893956	0.00000000
H	8.97870640	7.95502667	0.00000000
H	12.87502332	6.32538824	0.00000000
H	13.80925127	8.65811598	0.00000000

H	12.26778062	10.61763224	0.00000000
O	9.63018849	10.37740385	0.00000000
N	3.52849073	-0.26064229	-3.40000000
O	5.72209191	3.82344358	-3.40000000
N	4.54117224	1.83841331	-3.40000000
C	3.38396035	1.08648657	-3.40000000
N	2.16403483	1.62647301	-3.40000000
C	2.19121352	2.97694463	-3.40000000
C	3.30391005	3.84088446	-3.40000000
C	4.61013345	3.25499353	-3.40000000
N	1.09469962	3.81615206	-3.40000000
C	1.58271337	5.12552950	-3.40000000
N	2.89642836	5.17373573	-3.40000000
H	4.44590671	-0.72331780	-3.40000000
H	2.68146429	-0.81104040	-3.40000000
H	5.45850436	1.34473970	-3.40000000
H	0.12702485	3.51513525	-3.40000000
H	0.91527786	5.97855619	-3.40000000
O	6.12433817	-1.55688012	-3.40000000
N	8.17910809	2.56583589	-3.40000000
N	7.13018792	0.51883880	-3.40000000
C	9.56671161	0.57993165	-3.40000000
C	7.14082375	-0.84269401	-3.40000000
N	8.41185379	-1.47969708	-3.40000000
C	9.58402784	-0.77999742	-3.40000000
C	8.27542046	1.22826850	-3.40000000
H	10.48784455	1.15218876	-3.40000000
H	8.39857750	-2.49449952	-3.40000000
H	10.50044469	-1.36280207	-3.40000000
H	7.24421137	3.03781271	-3.40000000
H	9.01915542	3.12800464	-3.40000000
N	6.60546799	-0.85026063	3.40000000
O	3.39913155	2.49803001	3.40000000
N	4.92208327	0.76150058	3.40000000
C	5.27960996	-0.57143148	3.40000000
N	4.38907462	-1.56478460	3.40000000
C	3.11309847	-1.12161745	3.40000000
C	2.63528500	0.20359192	3.40000000
C	3.59614561	1.26483395	3.40000000
N	1.97612334	-1.90513479	3.40000000
C	0.88163594	-1.03638624	3.40000000
N	1.24174934	0.22792750	3.40000000
H	7.32899567	-0.12072078	3.40000000
H	6.86718213	-1.82591301	3.40000000
H	5.67506599	1.48138173	3.40000000
H	1.96337939	-2.91846749	3.40000000
H	-0.13588957	-1.40755539	3.40000000
O	8.64042440	1.21797748	3.40000000
N	5.35444730	4.44616911	3.40000000
N	6.97712306	2.81602986	3.40000000
C	7.67194753	5.15218032	3.40000000
C	8.27530436	2.40540836	3.40000000
N	9.27390017	3.41738499	3.40000000
C	8.97066795	4.74840784	3.40000000
C	6.65631164	4.12443656	3.40000000
H	7.41234441	6.20506698	3.40000000
H	10.23493204	3.09116728	3.40000000
H	9.80813648	5.43987551	3.40000000
H	4.61667169	3.70287835	3.40000000
H	5.07938194	5.41882130	3.40000000

G–C/^{O}Pu–^{N}Pz/G–C (-16367.9 kcal mol^{-1})

N	7.33013408	1.96989463	0.00000000
C	8.03077832	0.73654602	0.00000000

C	9.47693419	0.90332169	0.00000000
C	10.02565107	2.14854481	0.00000000
C	9.17267013	3.30836867	0.00000000
N	7.84183785	3.20028821	0.00000000
H	11.10334109	2.29449904	0.00000000
O	7.41086905	-0.33177688	0.00000000
C	9.71349992	4.61972503	0.00000000
H	6.27597901	1.91472201	0.00000000
H	10.08034174	0.00000000	0.00000000
C	10.20064400	5.73411584	0.00000000
C	10.78770958	7.02971808	0.00000000
N	9.95206334	8.09742898	0.00000000
C	12.18507505	7.18222845	0.00000000
C	10.53292896	9.33183002	0.00000000
C	12.66710962	8.49429183	0.00000000
N	11.87789930	9.56890332	0.00000000
H	10.15633508	11.31820660	0.00000000
H	12.84593479	6.32287885	0.00000000
H	13.74092050	8.68696863	0.00000000
N	9.72034031	10.40633795	0.00000000
H	8.69394184	10.30993790	0.00000000
N	4.50710312	1.63166986	0.00000000
C	4.08756272	0.35098259	0.00000000
N	2.81359046	-0.08841809	0.00000000
C	1.93349027	0.91676035	0.00000000
C	2.22872367	2.30492280	0.00000000
C	3.59582289	2.65144873	0.00000000
N	0.55178558	0.85079270	0.00000000
C	0.08963936	2.16234282	0.00000000
N	1.05844372	3.05691564	0.00000000
H	-0.97023376	2.38742616	0.00000000
H	4.87967424	-0.39607657	0.00000000
H	0.00000000	0.00000000	0.00000000
C	4.07791901	3.97364551	0.00000000
C	4.56180074	5.09134813	0.00000000
C	5.12470629	6.38248389	0.00000000
C	4.31230365	7.56797564	0.00000000
C	6.50563756	6.55184930	0.00000000
C	4.87960334	8.81142526	0.00000000
N	7.05507232	7.78594872	0.00000000
C	6.31542497	8.98991817	0.00000000
H	3.23105725	7.45272627	0.00000000
H	7.18656660	5.70645155	0.00000000
O	6.90470663	10.08913265	0.00000000
H	4.27917371	9.71601732	0.00000000
H	8.10137018	7.88169798	0.00000000
N	3.52849073	-0.26064229	-3.40000000
O	5.72209191	3.82344358	-3.40000000
N	4.54117224	1.83841331	-3.40000000
C	3.38396035	1.08648657	-3.40000000
N	2.16403483	1.62647301	-3.40000000
C	2.19121352	2.97694463	-3.40000000
C	3.30391005	3.84088446	-3.40000000
C	4.61013345	3.25499353	-3.40000000
N	1.09469962	3.81615206	-3.40000000
C	1.58271337	5.12552950	-3.40000000
N	2.89642836	5.17373573	-3.40000000
H	4.44590671	-0.72331780	-3.40000000
H	2.68146429	-0.81104040	-3.40000000
H	5.45850436	1.34473970	-3.40000000
H	0.12702485	3.51513525	-3.40000000
H	0.91527786	5.97855619	-3.40000000
O	6.12433817	-1.55688012	-3.40000000
N	8.17910809	2.56583589	-3.40000000
N	7.13018792	0.51883880	-3.40000000

C	9.56671161	0.57993165	-3.40000000
C	7.14082375	-0.84269401	-3.40000000
N	8.41185379	-1.47969708	-3.40000000
C	9.58402784	-0.77999742	-3.40000000
C	8.27542046	1.22826850	-3.40000000
H	10.48784455	1.15218876	-3.40000000
H	8.39857750	-2.49449952	-3.40000000
H	10.50044469	-1.36280207	-3.40000000
H	7.24421137	3.03781271	-3.40000000
H	9.01915542	3.12800464	-3.40000000
N	6.60546799	-0.85026063	3.40000000
O	3.39913155	2.49803001	3.40000000
N	4.92208327	0.76150058	3.40000000
C	5.27960996	-0.57143148	3.40000000
N	4.38907462	-1.56478460	3.40000000
C	3.11309847	-1.12161745	3.40000000
C	2.63528500	0.20359192	3.40000000
C	3.59614561	1.26483395	3.40000000
N	1.97612334	-1.90513479	3.40000000
C	0.88163594	-1.03638624	3.40000000
N	1.24174934	0.22792750	3.40000000
H	7.32899567	-0.12072078	3.40000000
H	6.86718213	-1.82591301	3.40000000
H	5.67506599	1.48138173	3.40000000
H	1.96337939	-2.91846749	3.40000000
H	-0.13588957	-1.40755539	3.40000000
O	8.64042440	1.21797748	3.40000000
N	5.35444730	4.44616911	3.40000000
N	6.97712306	2.81602986	3.40000000
C	7.67194753	5.15218032	3.40000000
C	8.27530436	2.40540836	3.40000000
N	9.27390017	3.41738499	3.40000000
C	8.97066795	4.74840784	3.40000000
C	6.65631164	4.12443656	3.40000000
H	7.41234441	6.20506698	3.40000000
H	10.23493204	3.09116728	3.40000000
H	9.80813648	5.43987551	3.40000000
H	4.61667169	3.70287835	3.40000000
H	5.07938194	5.41882130	3.40000000

G–C/^{N}Pu–^{O}Pz/^{N}Pu–^{O}Pz (-19524.3 kcal mol^{-1})

N	4.49675606	1.66092128	0.00000000
C	4.08556650	0.37769050	0.00000000
N	2.81422158	-0.07028460	0.00000000
C	1.92773825	0.92915896	0.00000000
C	2.21390905	2.31928955	0.00000000
C	3.57877754	2.67487234	0.00000000
N	0.54647006	0.85416665	0.00000000
C	0.07590335	2.16290401	0.00000000
N	1.03877555	3.06366951	0.00000000
H	-0.98542624	2.38114497	0.00000000
H	4.88207390	-0.36470859	0.00000000
H	0.00000000	0.00000000	0.00000000
C	4.05303118	3.99988188	0.00000000
C	4.53105457	5.12059361	0.00000000
C	5.11838295	6.39756338	0.00000000
C	4.35980477	7.59617086	0.00000000
C	6.52266165	6.56743619	0.00000000
N	4.90899138	8.80208311	0.00000000
N	7.10220035	7.76540514	0.00000000
C	6.27360050	8.85941986	0.00000000
H	3.27061377	7.54860508	0.00000000
H	7.17157811	5.69395009	0.00000000
H	7.87593978	10.18702456	0.00000000

H	6.23454293	10.87892744	0.00000000
N	6.84559113	10.07280239	0.00000000
N	7.33127993	1.98732974	0.00000000
C	8.02332540	0.74976602	0.00000000
C	9.47071273	0.90717484	0.00000000
C	10.02788061	2.14845224	0.00000000
C	9.18233083	3.31444223	0.00000000
N	7.85092171	3.21521674	0.00000000
H	11.10663545	2.28708103	0.00000000
O	7.39718555	-0.31516879	0.00000000
C	9.73680405	4.61964370	0.00000000
H	6.27794079	1.93974736	0.00000000
H	10.06839916	0.00000000	0.00000000
C	10.24779244	5.72437213	0.00000000
C	10.84597644	7.00548347	0.00000000
N	10.01370423	8.10497189	0.00000000
C	12.22140291	7.18957407	0.00000000
C	10.45655459	9.43784916	0.00000000
C	12.73102694	8.50949331	0.00000000
C	11.89278930	9.59893956	0.00000000
H	8.97870640	7.95502667	0.00000000
H	12.87502332	6.32538824	0.00000000
H	13.80925127	8.65811598	0.00000000
H	12.26778062	10.61763224	0.00000000
O	9.63018849	10.37740385	0.00000000
N	4.74124196	2.21572189	-3.40000000
C	3.65431849	1.41925754	-3.40000000
N	2.36246569	1.80411587	-3.40000000
C	2.23274379	3.13374452	-3.40000000
C	3.28135909	4.09017682	-3.40000000
C	4.59456722	3.57559977	-3.40000000
N	1.07119498	3.88496354	-3.40000000
C	1.45975503	5.22034648	-3.40000000
N	2.76819168	5.38311900	-3.40000000
H	0.72940017	6.02074101	-3.40000000
H	3.86233528	0.35046876	-3.40000000
H	0.12702485	3.51513525	-3.40000000
C	5.75706754	4.36879571	-3.40000000
C	6.80253441	4.99449545	-3.40000000
C	8.02827705	5.68236273	-3.40000000
C	8.11909821	7.09793762	-3.40000000
C	9.26421111	4.99437841	-3.40000000
N	9.27221695	7.75073733	-3.40000000
N	10.43721625	5.62291135	-3.40000000
C	10.40991061	6.99502662	-3.40000000
H	7.20996572	7.69966650	-3.40000000
H	9.27577331	3.90628979	-3.40000000
H	12.48657678	7.12724999	-3.40000000
H	11.56534915	8.65180004	-3.40000000
N	11.58586911	7.64046605	-3.40000000
N	7.22627802	0.81370056	-3.40000000
C	7.05873286	-0.59428364	-3.40000000
C	8.32221639	-1.31769016	-3.40000000
C	9.50257923	-0.64097071	-3.40000000
C	9.50386680	0.79933669	-3.40000000
N	8.36841092	1.50164423	-3.40000000
H	10.45679418	-1.16289385	-3.40000000
O	5.92622211	-1.08779823	-3.40000000
C	10.71962324	1.52935568	-3.40000000
H	6.34614053	1.39434282	-3.40000000
H	8.27253088	-2.40292129	-3.40000000
C	11.78236461	2.12274832	-3.40000000
C	13.01932398	2.80758543	-3.40000000
N	12.99226470	4.18628758	-3.40000000
C	14.24027311	2.14806246	-3.40000000

C	14.13398377	5.00430703	-3.40000000
C	15.42839668	2.91635007	-3.40000000
C	15.39060862	4.29043432	-3.40000000
H	12.06679828	4.67333581	-3.40000000
H	14.26110744	1.06473300	-3.40000000
H	16.38805670	2.40282397	-3.40000000
H	16.29275550	4.89415964	-3.40000000
O	14.01769594	6.25014855	-3.40000000
N	6.60546799	-0.85026063	3.40000000
O	3.39913155	2.49803001	3.40000000
N	4.92208327	0.76150058	3.40000000
C	5.27960996	-0.57143148	3.40000000
N	4.38907462	-1.56478460	3.40000000
C	3.11309847	-1.12161745	3.40000000
C	2.63528500	0.20359192	3.40000000
C	3.59614561	1.26483395	3.40000000
N	1.97612334	-1.90513479	3.40000000
C	0.88163594	-1.03638624	3.40000000
N	1.24174934	0.22792750	3.40000000
H	7.32899567	-0.12072078	3.40000000
H	6.86718213	-1.82591301	3.40000000
H	5.67506599	1.48138173	3.40000000
H	1.96337939	-2.91846749	3.40000000
H	-0.13588957	-1.40755539	3.40000000
O	8.64042440	1.21797748	3.40000000
N	5.35444730	4.44616911	3.40000000
N	6.97712306	2.81602986	3.40000000
C	7.67194753	5.15218032	3.40000000
C	8.27530436	2.40540836	3.40000000
N	9.27390017	3.41738499	3.40000000
C	8.97066795	4.74840784	3.40000000
C	6.65631164	4.12443656	3.40000000
H	7.41234441	6.20506698	3.40000000
H	10.23493204	3.09116728	3.40000000
H	9.80813648	5.43987551	3.40000000
H	4.61667169	3.70287835	3.40000000
H	5.07938194	5.41882130	3.40000000

^{N}Pu–^{O}Pz/^{N}Pu–^{O}Pz/^{N}Pu–^{O}Pz (-22678.0 kcal mol^{-1})

N	4.49675606	1.66092128	0.00000000
C	4.08556650	0.37769050	0.00000000
N	2.81422158	-0.07028460	0.00000000
C	1.92773825	0.92915896	0.00000000
C	2.21390905	2.31928955	0.00000000
C	3.57877754	2.67487234	0.00000000
N	0.54647006	0.85416665	0.00000000
C	0.07590335	2.16290401	0.00000000
N	1.03877555	3.06366951	0.00000000
H	-0.98542624	2.38114497	0.00000000
H	4.88207390	-0.36470859	0.00000000
H	0.00000000	0.00000000	0.00000000
C	4.05303118	3.99988188	0.00000000
C	4.53105457	5.12059361	0.00000000
C	5.11838295	6.39756338	0.00000000
C	4.35980477	7.59617086	0.00000000
C	6.52266165	6.56743619	0.00000000
N	4.90899138	8.80208311	0.00000000
N	7.10220035	7.76540514	0.00000000
C	6.27360050	8.85941986	0.00000000
H	3.27061377	7.54860508	0.00000000
H	7.17157811	5.69395009	0.00000000
H	7.87593978	10.18702456	0.00000000
H	6.23454293	10.87892744	0.00000000
N	6.84559113	10.07280239	0.00000000

N	7.33127993	1.98732974	0.00000000
C	8.02332540	0.74976602	0.00000000
C	9.47071273	0.90717484	0.00000000
C	10.02788061	2.14845224	0.00000000
C	9.18233083	3.31444223	0.00000000
N	7.85092171	3.21521674	0.00000000
H	11.10663545	2.28708103	0.00000000
O	7.39718555	-0.31516879	0.00000000
C	9.73680405	4.61964370	0.00000000
H	6.27794079	1.93974736	0.00000000
H	10.06839916	0.00000000	0.00000000
C	10.24779244	5.72437213	0.00000000
C	10.84597644	7.00548347	0.00000000
N	10.01370423	8.10497189	0.00000000
C	12.22140291	7.18957407	0.00000000
C	10.45655459	9.43784916	0.00000000
C	12.73102694	8.50949331	0.00000000
C	11.89278930	9.59893956	0.00000000
H	8.97870640	7.95502667	0.00000000
H	12.87502332	6.32538824	0.00000000
H	13.80925127	8.65811598	0.00000000
H	12.26778062	10.61763224	0.00000000
O	9.63018849	10.37740385	0.00000000
N	4.74124196	2.21572189	-3.40000000
C	3.65431849	1.41925754	-3.40000000
N	2.36246569	1.80411587	-3.40000000
C	2.23274380	3.13374452	-3.40000000
C	3.28135910	4.09017682	-3.40000000
C	4.59456722	3.57559977	-3.40000000
N	1.07119498	3.88496354	-3.40000000
C	1.45975504	5.22034648	-3.40000000
N	2.76819169	5.38311900	-3.40000000
H	0.72940018	6.02074100	-3.40000000
H	3.86233528	0.35046876	-3.40000000
H	0.12702486	3.51513525	-3.40000000
C	5.75706754	4.36879571	-3.40000000
C	6.80253441	4.99449544	-3.40000000
C	8.02827705	5.68236273	-3.40000000
C	8.11909821	7.09793762	-3.40000000
C	9.26421112	4.99437841	-3.40000000
N	9.27221695	7.75073733	-3.40000000
N	10.43721626	5.62291135	-3.40000000
C	10.40991062	6.99502662	-3.40000000
H	7.20996572	7.69966650	-3.40000000
H	9.27577331	3.90628979	-3.40000000
H	12.48657679	7.12724999	-3.40000000
H	11.56534915	8.65180004	-3.40000000
N	11.58586911	7.64046605	-3.40000000
N	7.22627802	0.81370056	-3.40000000
C	7.05873287	-0.59428365	-3.40000000
C	8.32221640	-1.31769016	-3.40000000
C	9.50257923	-0.64097071	-3.40000000
C	9.50386681	0.79933669	-3.40000000
N	8.36841092	1.50164423	-3.40000000
H	10.45679419	-1.16289385	-3.40000000
O	5.92622211	-1.08779824	-3.40000000
C	10.71962324	1.52935568	-3.40000000
H	6.34614054	1.39434281	-3.40000000
H	8.27253088	-2.40292129	-3.40000000
C	11.78236461	2.12274832	-3.40000000
C	13.01932399	2.80758543	-3.40000000
N	12.99226470	4.18628758	-3.40000000
C	14.24027312	2.14806246	-3.40000000
C	14.13398377	5.00430703	-3.40000000
C	15.42839668	2.91635007	-3.40000000

C	15.39060862	4.29043432	-3.40000000
H	12.06679828	4.67333581	-3.40000000
H	14.26110745	1.06473300	-3.40000000
H	16.38805670	2.40282397	-3.40000000
H	16.29275551	4.89415964	-3.40000000
O	14.01769594	6.25014855	-3.40000000
N	4.62506644	1.06837295	3.40000000
C	5.04667122	-0.21147372	3.40000000
N	4.28144473	-1.32117098	3.40000000
C	2.97680647	-1.03366598	3.40000000
C	2.39122525	0.25918026	3.40000000
C	3.28642073	1.34910235	3.40000000
N	1.90341640	-1.90622511	3.40000000
C	0.75346342	-1.12402652	3.40000000
N	1.00298672	0.17067016	3.40000000
H	-0.23344907	-1.57129975	3.40000000
H	6.12743048	-0.34391190	3.40000000
H	1.96337940	-2.91846749	3.40000000
C	2.89127892	2.69981688	3.40000000
C	2.61927014	3.88746682	3.40000000
C	2.34384478	5.26578002	3.40000000
C	1.02561834	5.78959278	3.40000000
C	3.38008138	6.22862432	3.40000000
N	0.76110221	7.08800007	3.40000000
N	3.14478956	7.53844586	3.40000000
C	1.83139248	7.93648359	3.40000000
H	0.17240278	5.11090085	3.40000000
H	4.41848807	5.90338275	3.40000000
H	2.34736573	9.95236975	3.40000000
H	0.61275747	9.54734208	3.40000000
N	1.58093426	9.25433834	3.40000000
N	6.72638634	2.99853427	3.40000000
C	8.01368459	2.40409831	3.40000000
C	9.09212295	3.38219765	3.40000000
C	8.81327669	4.71390722	3.40000000
C	7.44386082	5.16021125	3.40000000
N	6.42505150	4.29735349	3.40000000
H	9.60452373	5.46013645	3.40000000
O	8.13307978	1.17451218	3.40000000
C	7.12526091	6.54205260	3.40000000
H	5.90218530	2.34090210	3.40000000
H	10.10888542	2.99958905	3.40000000
C	6.88931612	7.73614811	3.40000000
C	6.62023879	9.12419269	3.40000000
N	5.30065335	9.52450018	3.40000000
C	7.62477644	10.08158051	3.40000000
C	4.87548121	10.86312145	3.40000000
C	7.26124188	11.44896710	3.40000000
C	5.94273294	11.83764390	3.40000000
H	4.55145810	8.79483549	3.40000000
H	8.66152214	9.76662793	3.40000000
H	8.04618547	12.20296972	3.40000000
H	5.64733476	12.88219796	3.40000000
O	3.65468060	11.13751136	3.40000000

S10. Optimized Geometries in Water

Cartesian coordinates (Å) and ADF total bonding energies (in kcal mol^{-1}) of the WC base pairs optimized at BLYP-D3(BJ)/TZ2P level of theory in water.

A–T (-4511.5 kcal mol^{-1})

N	4.59362061	-1.42324430	0.00000000
C	4.11472266	-0.16348963	0.00000000
N	2.83096765	0.23291518	0.00000000
C	1.98311285	-0.81977333	0.00000000
C	2.34262891	-2.17899152	0.00000000
C	3.72679637	-2.47701379	0.00000000
N	0.60290236	-0.81623453	0.00000000
C	0.19167759	-2.13329993	0.00000000
N	1.20650731	-2.98811796	0.00000000
N	4.22675705	-3.72662446	0.00000000
H	4.87194754	0.61925678	0.00000000
H	0.00000000	0.00000000	0.00000000
H	-0.85706123	-2.39959278	0.00000000
H	3.59899438	-4.51969988	0.00000000
H	5.24292768	-3.87537936	0.00000000
N	7.44111734	-1.76645429	0.00000000
C	8.19168180	-0.60815965	0.00000000
N	9.55914485	-0.82510648	0.00000000
C	10.12842843	-2.07708710	0.00000000
C	9.38072615	-3.21416744	0.00000000
C	7.93307577	-3.07206955	0.00000000
O	7.13335774	-4.03769629	0.00000000
O	7.69054235	0.52818334	0.00000000
C	9.98429120	-4.59244466	0.00000000
H	6.39589445	-1.64161619	0.00000000
H	10.15048679	0.00000000	0.00000000
H	11.21327702	-2.08908493	0.00000000
H	9.66357774	-5.16052698	0.88220861
H	11.07688581	-4.53571428	0.00000000
H	9.66357774	-5.16052698	-0.88220861

G–C (-4421.7 kcal mol^{-1})

N	5.01365243	-0.99199182	0.00000000
O	4.30260090	3.58796152	0.00000000
N	4.56716532	1.29195847	0.00000000
C	4.09292299	-0.00598124	0.00000000
N	2.78699383	-0.30650083	0.00000000
C	1.99120808	0.78584029	0.00000000
C	2.37604854	2.13811247	0.00000000
C	3.76721199	2.44848064	0.00000000
N	0.61326544	0.80857250	0.00000000
C	0.22151194	2.13729412	0.00000000
N	1.25137639	2.96774397	0.00000000
H	6.02751807	-0.80363867	0.00000000
H	4.68066827	-1.94733299	0.00000000
H	5.60211828	1.44276463	0.00000000
H	0.00000000	0.00000000	0.00000000
H	-0.82262775	2.42026635	0.00000000
O	7.83461905	-0.55738136	0.00000000
N	7.14844079	4.00206529	0.00000000
N	7.46163154	1.71656440	0.00000000
C	9.41590662	3.17447096	0.00000000
C	8.27003082	0.62421145	0.00000000
N	9.65603357	0.82777239	0.00000000
C	10.21432266	2.07245899	0.00000000
C	7.99620203	2.96060279	0.00000000
H	9.83388744	4.17428544	0.00000000
H	10.24447549	-0.00000000	0.00000000
H	11.29744761	2.11566317	0.00000000
H	6.12873984	3.85217674	0.00000000
H	7.51095075	4.94658058	0.00000000

Cartesian coordinates (Å) and ADF total bonding energies (in kcal mol^{-1}) of the UBPs optimized at BLYP-D3(BJ)/TZ2P level of theory in water.

^{N}Pu–^{O}Pz (-7565.7 kcal mol^{-1})

N	1.51981685	-3.05336573	0.00000000
C	0.80014078	-4.18950215	0.00000000
N	-0.54305023	-4.31069350	0.00000000
C	-1.16368205	-3.12398445	0.00000000
C	-0.53122393	-1.85256843	0.00000000
C	0.87533294	-1.84588885	0.00000000
N	-2.51321799	-2.85074180	0.00000000
C	-2.64757384	-1.47821041	0.00000000
N	-1.48438150	-0.83960168	0.00000000
H	-3.62161866	-1.00704914	0.00000000
H	1.38135433	-5.10952567	0.00000000
H	-3.26838944	-3.52903120	0.00000000
C	1.65359814	-0.67020371	0.00000000
C	2.36228083	0.32253661	0.00000000
C	3.21527763	1.44113020	0.00000000
C	2.73517222	2.77308386	0.00000000
C	4.62184345	1.29985082	0.00000000
N	3.53724010	3.83199785	0.00000000
N	5.44815746	2.34119972	0.00000000
C	4.88302967	3.58934941	0.00000000
H	1.66310169	2.96459088	0.00000000
H	5.06753119	0.30787371	0.00000000
H	6.73696505	4.51372016	0.00000000
H	5.31457707	5.57490735	0.00000000
N	5.71236479	4.64396039	0.00000000
N	4.38066630	-3.41268966	0.00000000
C	4.81316606	-4.74412162	0.00000000
C	6.25309964	-4.89323549	0.00000000
C	7.05348443	-3.79203436	0.00000000
C	6.45945290	-2.48333628	0.00000000
N	5.13698753	-2.30942685	0.00000000
H	8.13620494	-3.87759200	0.00000000
O	3.97408385	-5.67282639	0.00000000
C	7.27194258	-1.31966809	0.00000000
H	3.34453693	-3.23879075	0.00000000
H	6.65807775	-5.90076941	0.00000000
C	8.01513619	-0.35632593	0.00000000
C	8.90526454	0.74255143	0.00000000
N	8.36685849	2.01156058	0.00000000
C	10.28376124	0.58441682	0.00000000
C	9.11935670	3.18281708	0.00000000
C	11.09852503	1.73808814	0.00000000
C	10.54594803	2.99965295	0.00000000
H	7.32791181	2.11512625	0.00000000
H	10.70787509	-0.41247863	0.00000000
H	12.17966143	1.62171264	0.00000000
H	11.16203347	3.89368214	0.00000000
O	8.53493524	4.30627280	0.00000000

^{O}Pu–^{N}Pz (-7565.5 kcal mol^{-1})

N	1.08238573	-2.39404804	0.00000000
C	2.01981923	-1.35288256	0.00000000
C	1.42986776	-0.03129609	0.00000000
C	0.07574368	0.11108050	0.00000000
C	-0.75972151	-1.05735486	0.00000000
N	-0.24945554	-2.28989421	0.00000000
H	-0.39179252	1.09135537	0.00000000
O	3.24296348	-1.61656202	0.00000000
C	-2.17446834	-0.94173933	0.00000000
H	1.45026754	-3.38065852	0.00000000
H	2.10017450	0.82294357	0.00000000
C	-3.38205762	-0.79953666	0.00000000
C	-4.78955477	-0.59183951	0.00000000
N	-5.59047392	-1.68569890	0.00000000
C	-5.31382955	0.71190861	0.00000000

C	-6.93392798	-1.46338732	0.00000000
C	-6.70411060	0.82302183	0.00000000
N	-7.52722558	-0.23320131	0.00000000
H	-8.74394126	-2.40020929	0.00000000
H	-4.66741683	1.58163440	0.00000000
H	-7.17729337	1.80425972	0.00000000
N	-7.74274888	-2.54414587	0.00000000
H	-7.35540327	-3.49581213	0.00000000
N	2.17717205	-5.03912655	0.00000000
C	3.52038862	-5.10732406	0.00000000
N	4.28551796	-6.21756259	0.00000000
C	3.55672438	-7.34101848	0.00000000
C	2.13840726	-7.41497479	0.00000000
C	1.44192695	-6.19308079	0.00000000
N	3.98163276	-8.65048168	0.00000000
C	2.85218614	-9.44183329	0.00000000
N	1.72432259	-8.74272111	0.00000000
H	2.92039754	-10.52170488	0.00000000
H	4.03566848	-4.14884503	0.00000000
H	4.94348429	-8.97494479	0.00000000
C	0.03510800	-6.09400652	0.00000000
C	-1.17685404	-5.96754429	0.00000000
C	-2.57900093	-5.81490884	0.00000000
C	-3.46640339	-6.94093817	0.00000000
C	-3.15099862	-4.54678427	0.00000000
C	-4.82410502	-6.76519082	0.00000000
N	-4.49148629	-4.39108490	0.00000000
C	-5.41026341	-5.44988565	0.00000000
H	-3.04222339	-7.94132611	0.00000000
H	-2.55348816	-3.64198871	0.00000000
O	-6.64690966	-5.20648492	0.00000000
H	-5.50465020	-7.61117509	0.00000000
H	-4.88514549	-3.41893607	0.00000000

^{N}Pu–^{N}Pz (-7568.2 kcal mol^{-1})

N	3.37695275	7.04220591	2.35192988
C	3.37799932	8.38478357	2.43729587
N	4.29214759	9.16848847	3.04398034
C	5.28239200	8.46085367	3.60226445
C	5.40541315	7.04612805	3.58950233
C	4.39217510	6.32814610	2.92851434
N	6.37966961	8.90771462	4.30352493
C	7.10111841	7.79341463	4.67737859
N	6.55372042	6.65547136	4.26992569
H	8.01777737	7.88032467	5.24567784
H	2.53628880	8.88269973	1.95982289
H	6.61378850	9.87345610	4.51073330
C	4.37309858	4.92093645	2.83369429
C	4.35117186	3.70548747	2.73628591
C	4.30103002	2.30709927	2.58685091
C	5.38825763	1.46505988	2.91802988
C	3.14893568	1.64762460	2.09075268
N	5.36952686	0.14840332	2.72078669
N	3.09575075	0.33408167	1.90349496
C	4.22873606	-0.36960703	2.17999091
H	6.29478926	1.88811580	3.34795618
H	2.25673501	2.22214753	1.84786285
H	3.48789819	-2.06088302	1.29308207
H	5.07881557	-2.21233426	1.98214361
N	4.20783865	-1.69875892	1.91674443
N	1.10557032	5.95716691	0.98373018
C	0.08594161	6.86168973	0.66623610
C	-1.08469197	6.24387683	0.08064546
C	-1.12200029	4.89581039	-0.11148464
C	0.01081074	4.09620974	0.26039877
N	1.10648995	4.63252028	0.79786657
H	-1.99146079	4.40836094	-0.54290800
O	0.24010768	8.08246810	0.89762146
C	0.00560983	2.68329468	0.09558347

H	1.96872431	6.33274160	1.45396907
H	-1.91668122	6.88800976	-0.18776475
C	-0.01296237	1.47288627	-0.01280541
C	-0.01636034	0.04631357	-0.00542005
N	1.09682047	-0.58000181	-0.45066428
C	-1.11886563	-0.66598121	0.50006444
C	1.12477576	-1.92149610	-0.30836712
C	-0.99379056	-2.05245783	0.54994461
N	0.11943171	-2.70033284	0.16867921
H	2.26085231	-3.55361286	-0.77011684
H	-2.01246430	-0.15808521	0.84439331
H	-1.81073200	-2.66978838	0.92037537
N	2.30865268	-2.55221166	-0.61364108
H	2.94723623	-2.04337495	-1.21614456

^{O}Pu–^{O}Pz (-7544.7 kcal mol^{-1})

N	0.17660700	-2.11473093	-0.00179683
C	-0.20135573	-3.40469087	-0.04575984
N	-1.45306519	-3.89669524	0.05195380
C	-2.37058082	-2.93434144	0.20916056
C	-2.11303937	-1.53875323	0.27136027
C	-0.77057338	-1.13905163	0.15436525
N	-3.73566695	-3.05049725	0.34326339
C	-4.23872826	-1.77342117	0.47719917
N	-3.30104201	-0.83534802	0.43895036
H	-5.29900050	-1.59539093	0.59892147
H	0.60557407	-4.12293059	-0.17698690
H	-4.27314840	-3.91159856	0.34113537
C	-0.34448585	0.20593290	0.15881428
C	0.08038240	1.34652062	0.10819069
C	0.62591848	2.64459295	0.01950426
C	-0.15581177	3.83386179	0.20744160
C	1.97287699	2.79992571	-0.27747859
C	0.40872297	5.07612457	0.09936980
N	2.50912202	4.03632748	-0.38560829
C	1.80739772	5.24918890	-0.21420366
H	-1.21243450	3.73132692	0.43967401
H	2.63527477	1.95549288	-0.42409234
O	2.41639774	6.33362299	-0.34029674
H	-0.17828179	5.97809472	0.24279251
H	3.49938921	4.12895857	-0.60713419
N	3.01591541	-1.65790794	-0.14559957
C	3.79641117	-2.79571005	-0.38754518
C	5.21621941	-2.52615724	-0.47460908
C	5.68331019	-1.25785277	-0.30832369
C	4.75622925	-0.19266381	-0.04675272
N	3.44092643	-0.40238948	0.02427771
H	6.74415451	-1.03138064	-0.36264702
O	3.24784671	-3.91434608	-0.50230960
C	5.20653419	1.13785125	0.16360619
H	1.97389385	-1.78611852	-0.07928016
H	5.87907501	-3.36422271	-0.66882172
C	5.61725170	2.26618671	0.35861346
C	6.06457051	3.58840539	0.58127793
N	7.25029990	3.97385814	-0.01246471
C	5.37225079	4.50777753	1.35272238
C	7.83296348	5.24318900	0.09368745
C	5.89949686	5.81371279	1.49646147
C	7.08181859	6.17791252	0.89649149
H	7.75296358	3.29548775	-0.58150736
H	4.45115790	4.21320897	1.83989022
H	5.35392522	6.53642239	2.09780543
H	7.49228314	7.17731363	1.00402529
O	8.91851439	5.47787521	-0.49158178

Cartesian coordinates (Å) and ADF total bonding energies (in kcal mol^{-1}) of the stacked systems optimized at BLYP-D3(BJ)/TZ2P level of theory in water.

G–C/A–T/G–C (-13376.1 kcal mol^{-1})

N	5.01365243	-0.99199182	0.00000000
O	4.30260090	3.58796152	0.00000000

N	4.56716532	1.29195847	0.00000000
C	4.09292299	-0.00598124	0.00000000
N	2.78699383	-0.30650083	0.00000000
C	1.99120808	0.78584029	0.00000000
C	2.37604854	2.13811247	0.00000000
C	3.76721199	2.44848064	0.00000000
N	0.61326544	0.80857250	0.00000000
C	0.22151194	2.13729412	0.00000000
N	1.25137639	2.96774397	0.00000000
H	6.02751807	-0.80363867	0.00000000
H	4.68066827	-1.94733299	0.00000000
H	5.60211828	1.44276463	0.00000000
H	0.00000000	0.00000000	0.00000000
H	-0.82262775	2.42026635	0.00000000
O	7.83461905	-0.55738136	0.00000000
N	7.14844079	4.00206529	0.00000000
N	7.46163154	1.71656440	0.00000000
C	9.41590662	3.17447096	0.00000000
C	8.27003082	0.62421145	0.00000000
N	9.65603357	0.82777239	0.00000000
C	10.21432266	2.07245899	0.00000000
C	7.99620203	2.96060279	0.00000000
H	9.83388744	4.17428544	0.00000000
H	10.24447549	-0.00000000	0.00000000
H	11.29744761	2.11566317	0.00000000
H	6.12873984	3.85217674	0.00000000
H	7.51095075	4.94658058	0.00000000
N	7.79751313	0.32468251	6.80000000
O	3.22199166	1.06371573	6.80000000
N	5.48737522	0.60582748	6.80000000
C	6.57524030	-0.24628920	6.80000000
N	6.45749711	-1.58116730	6.80000000
C	5.17270765	-2.00045255	6.80000000
C	4.00554262	-1.21657244	6.80000000
C	4.14025810	0.20241166	6.80000000
N	4.72528034	-3.30392924	6.80000000
C	3.34053250	-3.26591140	6.80000000
N	2.86897337	-2.02982899	6.80000000
H	7.93168035	1.34713035	6.80000000
H	8.60319881	-0.28722091	6.80000000
H	5.66376809	1.63672791	6.80000000
H	5.30476904	-4.13704198	6.80000000
H	2.74875300	-4.17150403	6.80000000
O	8.25590065	3.14188321	6.80000000
N	3.70756846	3.89823540	6.80000000
N	5.97799023	3.48983889	6.80000000
C	5.19534291	5.79898285	6.80000000
C	7.26668874	3.92111667	6.80000000
N	7.50138919	5.30218741	6.80000000
C	6.49014270	6.21778120	6.80000000
C	4.96003078	4.38267465	6.80000000
H	4.37362601	6.50546589	6.80000000
H	8.47048607	5.60603319	6.80000000
H	6.78375710	7.26124506	6.80000000
H	3.53501602	2.88212406	6.80000000
H	2.92130277	4.53487414	6.80000000
N	4.86003644	0.92115831	3.40000000
C	5.21306508	-0.37949378	3.40000000
N	4.40748636	-1.45476427	3.40000000
C	3.10280263	-1.10147792	3.40000000
C	2.59472883	0.20947093	3.40000000
C	3.53937073	1.26416923	3.40000000
N	1.98826895	-1.91560824	3.40000000
C	0.88142950	-1.09179180	3.40000000
N	1.19999456	0.19627245	3.40000000
N	3.20934470	2.56899502	3.40000000
H	6.28575967	-0.56766331	3.40000000
H	1.98028131	-2.93033296	3.40000000
H	-0.12354104	-1.49278957	3.40000000
H	2.23531599	2.84161687	3.40000000
H	3.94400807	3.28663037	3.40000000
N	6.96197591	2.87253761	3.40000000
C	8.25002382	2.37662828	3.40000000
N	9.22880652	3.35591657	3.40000000
C	8.95347087	4.70340666	3.40000000

C	7.68020796	5.18383561	3.40000000
C	6.59255725	4.21796846	3.40000000
O	5.37799062	4.52911443	3.40000000
O	8.51251914	1.16274512	3.40000000
C	7.35837132	6.65365194	3.40000000
H	6.18975083	2.15717487	3.40000000
H	10.19219762	3.03597348	3.40000000
H	9.82407967	5.35077111	3.40000000
H	6.76499827	6.92472955	2.51779139
H	8.27564421	7.24996709	3.40000000
H	6.76499827	6.92472955	4.28220861

G–C/G–C/G–C (-13286.2 kcal mol^{-1})

N	6.61948949	-0.78592024	0.00000000
O	3.35220769	2.50139424	0.00000000
N	4.91580153	0.79939582	0.00000000
C	5.29504125	-0.52941211	0.00000000
N	4.41516335	-1.54014347	0.00000000
C	3.12929715	-1.12417207	0.00000000
C	2.64579398	0.19604265	0.00000000
C	3.58883902	1.26484114	0.00000000
N	2.00115648	-1.91571569	0.00000000
C	0.90321827	-1.07102424	0.00000000
N	1.24826994	0.20616293	0.00000000
H	7.32901282	-0.03760407	0.00000000
H	6.91163510	-1.75453066	0.00000000
H	5.66445443	1.52973065	0.00000000
H	1.98028131	-2.93033296	0.00000000
H	-0.10783539	-1.45582481	0.00000000
O	8.64624181	1.22380958	0.00000000
N	5.41113644	4.50915394	0.00000000
N	7.00789679	2.84423379	0.00000000
C	7.73200257	5.17239904	0.00000000
C	8.30397450	2.43566686	0.00000000
N	9.30562416	3.41502310	0.00000000
C	9.02568110	4.75014980	0.00000000
C	6.70914598	4.16489464	0.00000000
H	7.48247995	6.22694891	0.00000000
H	10.26823608	3.09121865	0.00000000
H	9.87655281	5.42174759	0.00000000
H	4.67428332	3.78852638	0.00000000
H	5.14924100	5.48636087	0.00000000
N	5.01365243	-0.99199182	-3.40000000
O	4.30260090	3.58796152	-3.40000000
N	4.56716532	1.29195847	-3.40000000
C	4.09292299	-0.00598124	-3.40000000
N	2.78699383	-0.30650083	-3.40000000
C	1.99120808	0.78584029	-3.40000000
C	2.37604854	2.13811247	-3.40000000
C	3.76721199	2.44848064	-3.40000000
N	0.61326544	0.80857250	-3.40000000
C	0.22151194	2.13729412	-3.40000000
N	1.25137639	2.96774397	-3.40000000
H	6.02751807	-0.80363867	-3.40000000
H	4.68066827	-1.94733299	-3.40000000
H	5.60211828	1.44276463	-3.40000000
H	0.00000000	0.00000000	-3.40000000
H	-0.82262775	2.42026635	-3.40000000
O	7.83461905	-0.55738136	-3.40000000
N	7.14844079	4.00206529	-3.40000000
N	7.46163154	1.71656440	-3.40000000
C	9.41590662	3.17447096	-3.40000000
C	8.27003082	0.62421145	-3.40000000
N	9.65603357	0.82777239	-3.40000000
C	10.21432266	2.07245899	-3.40000000
C	7.99620203	2.96060279	-3.40000000
H	9.83388744	4.17428544	-3.40000000
H	10.24447549	-0.00000000	-3.40000000
H	11.29744761	2.11566317	-3.40000000
H	6.12873984	3.85217674	-3.40000000
H	7.51095075	4.94658058	-3.40000000
N	7.79751313	0.32468251	3.40000000
O	3.22199166	1.06371573	3.40000000
N	5.48737522	0.60582748	3.40000000

C	6.57524030	-0.24628920	3.40000000
N	6.45749711	-1.58116730	3.40000000
C	5.17270765	-2.00045255	3.40000000
C	4.00554262	-1.21657244	3.40000000
C	4.14025810	0.20241166	3.40000000
N	4.72528034	-3.30392924	3.40000000
C	3.34053250	-3.26591140	3.40000000
N	2.86897337	-2.02982899	3.40000000
H	7.93168035	1.34713035	3.40000000
H	8.60319881	-0.28722091	3.40000000
H	5.66376809	1.63672791	3.40000000
H	5.30476904	-4.13704198	3.40000000
H	2.74875300	-4.17150403	3.40000000
O	8.25590065	3.14188321	3.40000000
N	3.70756846	3.89823540	3.40000000
N	5.97799023	3.48983889	3.40000000
C	5.19534291	5.79898285	3.40000000
C	7.26668874	3.92111667	3.40000000
N	7.50138919	5.30218741	3.40000000
C	6.49014270	6.21778120	3.40000000
C	4.96003078	4.38267465	3.40000000
H	4.37362601	6.50546589	3.40000000
H	8.47048607	5.60603319	3.40000000
H	6.78375710	7.26124506	3.40000000
H	3.53501602	2.88212406	3.40000000
H	2.92130277	4.53487414	3.40000000

G–C/^{N}Pu–^{O}Pz/G–C (-16433.3 kcal mol^{-1})

N	4.73256059	1.01658176	0.00000000
C	5.12769777	-0.26895556	0.00000000
N	4.34632569	-1.36818579	0.00000000
C	3.04120208	-1.06799486	0.00000000
C	2.48657744	0.23925166	0.00000000
C	3.39614928	1.31215702	0.00000000
N	1.95605239	-1.91555545	0.00000000
C	0.82597174	-1.12510518	0.00000000
N	1.09721332	0.17384268	0.00000000
H	-0.16536496	-1.55870383	0.00000000
H	6.20458763	-0.42567410	0.00000000
H	1.98028131	-2.93033296	0.00000000
C	3.00906587	2.66792201	0.00000000
C	2.71571807	3.85186101	0.00000000
C	2.42060309	5.22727452	0.00000000
C	1.09651767	5.72867210	0.00000000
C	3.44258524	6.20397291	0.00000000
N	0.81362359	7.02658684	0.00000000
N	3.18880198	7.50888534	0.00000000
C	1.87309468	7.89119591	0.00000000
H	0.25389091	5.03875159	0.00000000
H	4.48600946	5.89750587	0.00000000
H	2.37641447	9.90072492	0.00000000
H	0.64528928	9.51019788	0.00000000
N	1.61120076	9.20702744	0.00000000
N	6.86587303	2.95631146	0.00000000
C	8.15860544	2.41908710	0.00000000
C	9.20823761	3.41604044	0.00000000
C	8.89212351	4.74017473	0.00000000
C	7.51162225	5.13990011	0.00000000
N	6.51953932	4.24831131	0.00000000
H	9.66118589	5.50708207	0.00000000
O	8.31851009	1.17772413	0.00000000
C	7.15592339	6.51385115	0.00000000
H	6.05999473	2.28224539	0.00000000
H	10.23700796	3.06853010	0.00000000
C	6.90735091	7.70489128	0.00000000
C	6.65136001	9.09569270	0.00000000
N	5.33718221	9.51186813	0.00000000
C	7.66789449	10.04010647	0.00000000

C	4.93670765	10.84517810	0.00000000
C	7.32126904	11.40928445	0.00000000
C	6.00353171	11.80985336	0.00000000
H	4.58290408	8.78992592	0.00000000
H	8.70102625	9.71405147	0.00000000
H	8.11271365	12.15494832	0.00000000
H	5.72496128	12.85925698	0.00000000
O	3.70318425	11.13174611	0.00000000
N	5.01365243	-0.99199182	-3.40000000
O	4.30260090	3.58796152	-3.40000000
N	4.56716532	1.29195847	-3.40000000
C	4.09292299	-0.00598124	-3.40000000
N	2.78699383	-0.30650083	-3.40000000
C	1.99120808	0.78584029	-3.40000000
C	2.37604854	2.13811247	-3.40000000
C	3.76721199	2.44848064	-3.40000000
N	0.61326544	0.80857250	-3.40000000
C	0.22151194	2.13729412	-3.40000000
N	1.25137639	2.96774397	-3.40000000
H	6.02751807	-0.80363867	-3.40000000
H	4.68066827	-1.94733299	-3.40000000
H	5.60211828	1.44276463	-3.40000000
H	0.00000000	0.00000000	-3.40000000
H	-0.82262775	2.42026635	-3.40000000
O	7.83461905	-0.55738136	-3.40000000
N	7.14844079	4.00206529	-3.40000000
N	7.46163154	1.71656440	-3.40000000
C	9.41590662	3.17447096	-3.40000000
C	8.27003082	0.62421145	-3.40000000
N	9.65603357	0.82777239	-3.40000000
C	10.21432266	2.07245899	-3.40000000
C	7.99620203	2.96060279	-3.40000000
H	9.83388744	4.17428544	-3.40000000
H	10.24447549	-0.00000000	-3.40000000
H	11.29744761	2.11566317	-3.40000000
H	6.12873984	3.85217674	-3.40000000
H	7.51095075	4.94658058	-3.40000000
N	7.79751313	0.32468251	3.40000000
O	3.22199166	1.06371573	3.40000000
N	5.48737522	0.60582748	3.40000000
C	6.57524030	-0.24628920	3.40000000
N	6.45749711	-1.58116730	3.40000000
C	5.17270765	-2.00045255	3.40000000
C	4.00554262	-1.21657244	3.40000000
C	4.14025810	0.20241166	3.40000000
N	4.72528034	-3.30392924	3.40000000
C	3.34053250	-3.26591140	3.40000000
N	2.86897337	-2.02982899	3.40000000
H	7.93168035	1.34713035	3.40000000
H	8.60319881	-0.28722091	3.40000000
H	5.66376809	1.63672791	3.40000000
H	5.30476904	-4.13704198	3.40000000
H	2.74875300	-4.17150403	3.40000000
O	8.25590065	3.14188321	3.40000000
N	3.70756846	3.89823540	3.40000000
N	5.97799023	3.48983889	3.40000000
C	5.19534291	5.79898285	3.40000000
C	7.26668874	3.92111667	3.40000000
N	7.50138919	5.30218741	3.40000000
C	6.49014270	6.21778120	3.40000000
C	4.96003078	4.38267465	3.40000000
H	4.37362601	6.50546589	3.40000000
H	8.47048607	5.60603319	3.40000000
H	6.78375710	7.26124506	3.40000000
H	3.53501602	2.88212406	3.40000000

H	2.92130277	4.53487414	3.40000000

G–C/^{O}Pu–^{N}Pz/G–C (-16433.3 kcal mol^{-1})

N	6.86243207	2.93318885	0.00000000
C	8.15975029	2.40425354	0.00000000
C	9.20306080	3.40731720	0.00000000
C	8.87792379	4.72951576	0.00000000
C	7.49544273	5.11937394	0.00000000
N	6.50889037	4.22146578	0.00000000
H	9.64163787	5.50170079	0.00000000
O	8.32636289	1.16415316	0.00000000
C	7.12457689	6.48953235	0.00000000
H	6.05897228	2.25260849	0.00000000
H	10.23396082	3.06631622	0.00000000
C	6.84895519	7.67381532	0.00000000
C	6.56717549	9.06837136	0.00000000
N	5.26656578	9.45101251	0.00000000
C	7.61597011	10.00360023	0.00000000
C	5.02024720	10.79027290	0.00000000
C	7.24916311	11.34921539	0.00000000
N	5.97643580	11.76549750	0.00000000
H	3.52526678	12.17548661	0.00000000
H	8.65333847	9.69032838	0.00000000
H	8.01186885	12.12703908	0.00000000
N	3.72928358	11.18478935	0.00000000
H	2.96529724	10.49774484	0.00000000
N	4.74516169	1.00649128	0.00000000
C	5.13638679	-0.28029719	0.00000000
N	4.35129730	-1.37651204	0.00000000
C	3.04729624	-1.07175894	0.00000000
C	2.49687508	0.23748875	0.00000000
C	3.41028124	1.30697412	0.00000000
N	1.95943732	-1.91544370	0.00000000
C	0.83204113	-1.12117368	0.00000000
N	1.10737633	0.17691128	0.00000000
H	-0.16075232	-1.55144871	0.00000000
H	6.21279489	-0.44011265	0.00000000
H	1.98028131	-2.93033296	0.00000000
C	3.02654151	2.66406627	0.00000000
C	2.73462908	3.84712663	0.00000000
C	2.40286245	5.21798197	0.00000000
C	1.04266800	5.67107826	0.00000000
C	3.40196398	6.18603021	0.00000000
C	0.74771256	7.00795602	0.00000000
N	3.09398382	7.49991670	0.00000000
C	1.78639971	8.00532172	0.00000000
H	0.24533317	4.93286293	0.00000000
H	4.45574611	5.93065403	0.00000000
O	1.59613183	9.25124948	0.00000000
H	-0.27889206	7.36138788	0.00000000
H	3.87509924	8.19984499	0.00000000
N	5.01365243	-0.99199182	-3.40000000
O	4.30260090	3.58796152	-3.40000000
N	4.56716532	1.29195847	-3.40000000
C	4.09292299	-0.00598124	-3.40000000
N	2.78699383	-0.30650083	-3.40000000
C	1.99120808	0.78584029	-3.40000000
C	2.37604854	2.13811247	-3.40000000
C	3.76721199	2.44848064	-3.40000000
N	0.61326544	0.80857250	-3.40000000
C	0.22151194	2.13729412	-3.40000000
N	1.25137639	2.96774397	-3.40000000
H	6.02751807	-0.80363867	-3.40000000
H	4.68066827	-1.94733299	-3.40000000

H	5.60211828	1.44276463	-3.40000000
H	0.00000000	0.00000000	-3.40000000
H	-0.82262775	2.42026635	-3.40000000
O	7.83461905	-0.55738136	-3.40000000
N	7.14844079	4.00206529	-3.40000000
N	7.46163154	1.71656440	-3.40000000
C	9.41590662	3.17447096	-3.40000000
C	8.27003082	0.62421145	-3.40000000
N	9.65603357	0.82777239	-3.40000000
C	10.21432266	2.07245899	-3.40000000
C	7.99620203	2.96060279	-3.40000000
H	9.83388744	4.17428544	-3.40000000
H	10.24447549	-0.00000000	-3.40000000
H	11.29744761	2.11566317	-3.40000000
H	6.12873984	3.85217674	-3.40000000
H	7.51095075	4.94658058	-3.40000000
N	7.79751313	0.32468251	3.40000000
O	3.22199166	1.06371573	3.40000000
N	5.48737522	0.60582748	3.40000000
C	6.57524030	-0.24628920	3.40000000
N	6.45749711	-1.58116730	3.40000000
C	5.17270765	-2.00045255	3.40000000
C	4.00554262	-1.21657244	3.40000000
C	4.14025810	0.20241166	3.40000000
N	4.72528034	-3.30392924	3.40000000
C	3.34053250	-3.26591140	3.40000000
N	2.86897337	-2.02982899	3.40000000
H	7.93168035	1.34713035	3.40000000
H	8.60319881	-0.28722091	3.40000000
H	5.66376809	1.63672791	3.40000000
H	5.30476904	-4.13704198	3.40000000
H	2.74875300	-4.17150403	3.40000000
O	8.25590065	3.14188321	3.40000000
N	3.70756846	3.89823540	3.40000000
N	5.97799023	3.48983889	3.40000000
C	5.19534291	5.79898285	3.40000000
C	7.26668874	3.92111667	3.40000000
N	7.50138919	5.30218741	3.40000000
C	6.49014270	6.21778120	3.40000000
C	4.96003078	4.38267465	3.40000000
H	4.37362601	6.50546589	3.40000000
H	8.47048607	5.60603319	3.40000000
H	6.78375710	7.26124506	3.40000000
H	3.53501602	2.88212406	3.40000000
H	2.92130277	4.53487414	3.40000000

G–C/^{N}Pu–^{O}Pz/^{N}Pu–^{O}Pz (-19587.4 kcal mol^{-1})

N	4.54657898	1.57537191	0.00000000
C	4.11063179	0.30309456	0.00000000
N	2.83237718	-0.12692238	0.00000000
C	1.95295780	0.88306959	0.00000000
C	2.27263727	2.26665443	0.00000000
C	3.63913429	2.60002018	0.00000000
N	0.57686965	0.83521365	0.00000000
C	0.12723021	2.13894609	0.00000000
N	1.11017165	3.03038519	0.00000000
H	-0.92964093	2.37085051	0.00000000
H	4.88973714	-0.45667337	0.00000000
H	0.00000000	0.00000000	0.00000000
C	4.12287590	3.92437901	0.00000000
C	4.58145443	5.05463130	0.00000000
C	5.15114918	6.34082843	0.00000000
C	4.37465567	7.52474548	0.00000000
C	6.55203901	6.53028800	0.00000000

N	4.90868470	8.74106153	0.00000000
N	7.11373232	7.73515438	0.00000000
C	6.27401927	8.81780348	0.00000000
H	3.28743120	7.46187165	0.00000000
H	7.21605013	5.66904159	0.00000000
H	7.86238506	10.14770265	0.00000000
H	6.23232931	10.84928950	0.00000000
N	6.83556901	10.03627094	0.00000000
N	7.41260951	1.89071662	0.00000000
C	8.14267944	0.69624393	0.00000000
C	9.57784417	0.88583781	0.00000000
C	10.10040910	2.14289216	0.00000000
C	9.21851281	3.27771507	0.00000000
N	7.89183810	3.13953630	0.00000000
H	11.17337046	2.31128972	0.00000000
O	7.54239017	-0.40202940	0.00000000
C	9.73833454	4.59833936	0.00000000
H	6.36443417	1.81906909	0.00000000
H	10.20587542	0.00000000	0.00000000
C	10.23731100	5.70801830	0.00000000
C	10.84770258	6.98366796	0.00000000
N	10.02913218	8.09281529	0.00000000
C	12.22520873	7.15021078	0.00000000
C	10.48884140	9.40687875	0.00000000
C	12.74956548	8.46164036	0.00000000
C	11.91894208	9.56025398	0.00000000
H	8.99456137	7.95210533	0.00000000
H	12.86937956	6.27916713	0.00000000
H	13.82814784	8.59969563	0.00000000
H	12.31039784	10.57297894	0.00000000
O	9.65934045	10.36376400	0.00000000
N	4.73256060	1.01658176	3.40000000
C	5.12769777	-0.26895557	3.40000000
N	4.34632569	-1.36818579	3.40000000
C	3.04120208	-1.06799486	3.40000000
C	2.48657744	0.23925166	3.40000000
C	3.39614928	1.31215702	3.40000000
N	1.95605239	-1.91555545	3.40000000
C	0.82597174	-1.12510518	3.40000000
N	1.09721332	0.17384268	3.40000000
H	-0.16536497	-1.55870384	3.40000000
H	6.20458763	-0.42567410	3.40000000
H	1.98028131	-2.93033296	3.40000000
C	3.00906587	2.66792200	3.40000000
C	2.71571807	3.85186101	3.40000000
C	2.42060310	5.22727452	3.40000000
C	1.09651767	5.72867210	3.40000000
C	3.44258524	6.20397291	3.40000000
N	0.81362360	7.02658684	3.40000000
N	3.18880198	7.50888533	3.40000000
C	1.87309468	7.89119591	3.40000000
H	0.25389091	5.03875159	3.40000000
H	4.48600946	5.89750587	3.40000000
H	2.37641448	9.90072492	3.40000000
H	0.64528927	9.51019788	3.40000000
N	1.61120076	9.20702744	3.40000000
N	6.86587303	2.95631147	3.40000000
C	8.15860544	2.41908710	3.40000000
C	9.20823761	3.41604043	3.40000000
C	8.89212351	4.74017472	3.40000000
C	7.51162226	5.13990011	3.40000000
N	6.51953931	4.24831131	3.40000000
H	9.66118588	5.50708208	3.40000000
O	8.31851009	1.17772413	3.40000000
C	7.15592339	6.51385115	3.40000000

H	6.05999473	2.28224539	3.40000000
H	10.23700797	3.06853010	3.40000000
C	6.90735091	7.70489128	3.40000000
C	6.65136001	9.09569270	3.40000000
N	5.33718221	9.51186813	3.40000000
C	7.66789448	10.04010647	3.40000000
C	4.93670765	10.84517810	3.40000000
C	7.32126904	11.40928445	3.40000000
C	6.00353171	11.80985336	3.40000000
H	4.58290408	8.78992592	3.40000000
H	8.70102624	9.71405147	3.40000000
H	8.11271365	12.15494832	3.40000000
H	5.72496128	12.85925698	3.40000000
O	3.70318425	11.13174610	3.40000000
N	7.79751313	0.32468251	6.80000000
O	3.22199166	1.06371573	6.80000000
N	5.48737522	0.60582748	6.80000000
C	6.57524030	-0.24628920	6.80000000
N	6.45749711	-1.58116730	6.80000000
C	5.17270765	-2.00045255	6.80000000
C	4.00554262	-1.21657244	6.80000000
C	4.14025810	0.20241166	6.80000000
N	4.72528034	-3.30392924	6.80000000
C	3.34053250	-3.26591140	6.80000000
N	2.86897337	-2.02982899	6.80000000
H	7.93168035	1.34713035	6.80000000
H	8.60319881	-0.28722091	6.80000000
H	5.66376809	1.63672791	6.80000000
H	5.30476904	-4.13704198	6.80000000
H	2.74875300	-4.17150403	6.80000000
O	8.25590065	3.14188321	6.80000000
N	3.70756846	3.89823540	6.80000000
N	5.97799023	3.48983889	6.80000000
C	5.19534291	5.79898285	6.80000000
C	7.26668874	3.92111667	6.80000000
N	7.50138919	5.30218741	6.80000000
C	6.49014270	6.21778120	6.80000000
C	4.96003078	4.38267465	6.80000000
H	4.37362601	6.50546589	6.80000000
H	8.47048607	5.60603319	6.80000000
H	6.78375710	7.26124506	6.80000000
H	3.53501602	2.88212406	6.80000000
H	2.92130277	4.53487414	6.80000000

^{N}Pu–^{O}Pz/^{N}Pu–^{O}Pz/^{N}Pu–^{O}Pz (-22739.9 kcal mol^{-1})

N	4.73256060	1.01658176	0.00000000
C	5.12769777	-0.26895557	0.00000000
N	4.34632569	-1.36818579	0.00000000
C	3.04120208	-1.06799486	0.00000000
C	2.48657744	0.23925166	0.00000000
C	3.39614928	1.31215702	0.00000000
N	1.95605239	-1.91555545	0.00000000
C	0.82597174	-1.12510518	0.00000000
N	1.09721332	0.17384268	0.00000000
H	-0.16536497	-1.55870384	0.00000000
H	6.20458763	-0.42567410	0.00000000
H	1.98028131	-2.93033296	0.00000000
C	3.00906587	2.66792200	0.00000000
C	2.71571807	3.85186101	0.00000000
C	2.42060310	5.22727452	0.00000000
C	1.09651767	5.72867210	0.00000000
C	3.44258524	6.20397291	0.00000000
N	0.81362360	7.02658684	0.00000000
N	3.18880198	7.50888533	0.00000000

C	1.87309468	7.89119591	0.00000000
H	0.25389091	5.03875159	0.00000000
H	4.48600946	5.89750587	0.00000000
H	2.37641448	9.90072492	0.00000000
H	0.64528927	9.51019788	0.00000000
N	1.61120076	9.20702744	0.00000000
N	6.86587303	2.95631147	0.00000000
C	8.15860544	2.41908710	0.00000000
C	9.20823761	3.41604043	0.00000000
C	8.89212351	4.74017472	0.00000000
C	7.51162226	5.13990011	0.00000000
N	6.51953931	4.24831131	0.00000000
H	9.66118588	5.50708208	0.00000000
O	8.31851009	1.17772413	0.00000000
C	7.15592339	6.51385115	0.00000000
H	6.05999473	2.28224539	0.00000000
H	10.23700797	3.06853010	0.00000000
C	6.90735091	7.70489128	0.00000000
C	6.65136001	9.09569270	0.00000000
N	5.33718221	9.51186813	0.00000000
C	7.66789448	10.04010647	0.00000000
C	4.93670765	10.84517810	0.00000000
C	7.32126904	11.40928445	0.00000000
C	6.00353171	11.80985336	0.00000000
H	4.58290408	8.78992592	0.00000000
H	8.70102624	9.71405147	0.00000000
H	8.11271365	12.15494832	0.00000000
H	5.72496128	12.85925698	0.00000000
O	3.70318425	11.13174610	0.00000000
N	4.54657898	1.57537191	-3.40000000
C	4.11063179	0.30309456	-3.40000000
N	2.83237718	-0.12692238	-3.40000000
C	1.95295780	0.88306959	-3.40000000
C	2.27263727	2.26665443	-3.40000000
C	3.63913429	2.60002018	-3.40000000
N	0.57686965	0.83521365	-3.40000000
C	0.12723021	2.13894609	-3.40000000
N	1.11017165	3.03038519	-3.40000000
H	-0.92964093	2.37085051	-3.40000000
H	4.88973714	-0.45667337	-3.40000000
H	0.00000000	0.00000000	-3.40000000
C	4.12287590	3.92437901	-3.40000000
C	4.58145443	5.05463130	-3.40000000
C	5.15114918	6.34082843	-3.40000000
C	4.37465567	7.52474548	-3.40000000
C	6.55203901	6.53028800	-3.40000000
N	4.90868470	8.74106153	-3.40000000
N	7.11373232	7.73515438	-3.40000000
C	6.27401927	8.81780348	-3.40000000
H	3.28743120	7.46187165	-3.40000000
H	7.21605013	5.66904159	-3.40000000
H	7.86238506	10.14770265	-3.40000000
H	6.23232931	10.84928950	-3.40000000
N	6.83556901	10.03627094	-3.40000000
N	7.41260951	1.89071662	-3.40000000
C	8.14267944	0.69624393	-3.40000000
C	9.57784417	0.88583781	-3.40000000
C	10.10040910	2.14289216	-3.40000000
C	9.21851281	3.27771507	-3.40000000
N	7.89183810	3.13953630	-3.40000000
H	11.17337046	2.31128972	-3.40000000
O	7.54239017	-0.40202940	-3.40000000
C	9.73833454	4.59833936	-3.40000000
H	6.36443417	1.81906909	-3.40000000
H	10.20587542	0.00000000	-3.40000000

C	10.23731100	5.70801830	-3.40000000
C	10.84770258	6.98366796	-3.40000000
N	10.02913218	8.09281529	-3.40000000
C	12.22520873	7.15021078	-3.40000000
C	10.48884140	9.40687875	-3.40000000
C	12.74956548	8.46164036	-3.40000000
C	11.91894208	9.56025398	-3.40000000
H	8.99456137	7.95210533	-3.40000000
H	12.86937956	6.27916713	-3.40000000
H	13.82814784	8.59969563	-3.40000000
H	12.31039784	10.57297894	-3.40000000
O	9.65934045	10.36376400	-3.40000000
N	5.21147149	0.67382828	3.40000000
C	6.28676407	-0.13393746	3.40000000
N	6.30073208	-1.48251238	3.40000000
C	5.06841710	-2.00678522	3.40000000
C	3.85133611	-1.27520075	3.40000000
C	3.95655725	0.12743082	3.40000000
N	4.68869618	-3.33031113	3.40000000
C	3.30982672	-3.35506816	3.40000000
N	2.76576337	-2.14476547	3.40000000
H	2.76268137	-4.28854994	3.40000000
H	7.25010310	0.37225456	3.40000000
H	5.30476904	-4.13704198	3.40000000
C	2.84650153	0.99674582	3.40000000
C	1.91327628	1.78214708	3.40000000
C	0.86607548	2.72141576	3.40000000
C	-0.49984623	2.34877704	3.40000000
C	1.11878749	4.11228739	3.40000000
N	-1.49160749	3.23253116	3.40000000
N	0.14646424	5.01881366	3.40000000
C	-1.14268184	4.55475606	3.40000000
H	-0.77602051	1.29533604	3.40000000
H	2.14307222	4.47765971	3.40000000
H	-1.91665909	6.47634314	3.40000000
H	-3.08762276	5.14287026	3.40000000
N	-2.12798484	5.46534875	3.40000000
N	5.79721299	3.49703217	3.40000000
C	7.15882804	3.82225757	3.40000000
C	7.42200384	5.24576807	3.40000000
C	6.38795555	6.13120801	3.40000000
C	5.03615389	5.64315436	3.40000000
N	4.75760468	4.33871214	3.40000000
H	6.55936325	7.20369261	3.40000000
O	8.01784846	2.91196343	3.40000000
C	3.94079930	6.54562955	3.40000000
H	5.54144985	2.47801788	3.40000000
H	8.45855799	5.56932235	3.40000000
C	3.03962412	7.36309402	3.40000000
C	2.01503057	8.33780833	3.40000000
N	0.70721661	7.90204699	3.40000000
C	2.28231174	9.69935909	3.40000000
C	-0.40047405	8.74532438	3.40000000
C	1.19710324	10.60330602	3.40000000
C	-0.10441715	10.15282651	3.40000000
H	0.52133977	6.87462991	3.40000000
H	3.30978322	10.04283467	3.40000000
H	1.39910516	11.67176023	3.40000000
H	-0.94660934	10.83807228	3.40000000
O	-1.56685589	8.25211590	3.40000000

REFERENCES

1. Fonseca Guerra, C.; Bickelhaupt, F. M.; Snijders, J. G.; Baerends, E. J., Hydrogen Bonding in DNA Base Pairs: Reconciliation of Theory and Experiment, *J. Am. Chem. Soc.* **2000**, 122, 4117-4128.

2. Popelier, P. L. A., Non-covalent Interactions from a Quantum Chemical Topology Perspective. *J. Mol. Model.* **2022**, 28, 276.

3. Guillaumes, L.; Simon, S.; Fonseca Guerra, C., The Role of Aromaticity, Hybridization, Electrostatics, and Covalency in Resonance-Assisted Hydrogen Bonds of Adenine–Thymine (AT) Base Pairs and Their Mimics. *Chem. Eur. J.* **2015**, 4, 318–327.

4. Nieuwland, C.; Almacellas, D.; Veldhuizen, M. M.; de Azevedo Santos, L.; Poater, J.; Fonseca Guerra, C., Multiple Hydrogen-Bonded Dimers: Are Only the Frontier Atoms Relevant? *Phys. Chem. Chem. Phys.* **2024**, 26, 11306-11310.

5. Zgarbová, M.; Jurečka, P.; Lankaš, F.; Cheatham, T. E., III; Šponer, J.; Otyepka, M., Influence of BII Backbone Substates on DNA Twist: A Unified View and Comparison of Simulation and Experiment for All 136 Distinct Tetranucleotide Sequences. *J. Chem. Inf. Model.* **2017**, 57, 275–287.
